# Comparative phytolith profiles of the weed grasses *Echinochloa colona* and *Echinochloa crus-galli* (Poaceae): implications for species taxonomy

**DOI:** 10.3389/fpls.2026.1964606

**Published:** 2026-10-28

**Authors:** Sheikh Abdul Shakoor, Mudassir Ahmad Bhat, Davinder Kumar, Mayank Dhar Dwivedi

**Affiliations:** 1 Department of Botanical and Environmental Sciences, Guru Nanak Dev University, Amritsar (Punjab), India; 2 Department of Botany, Government Degree College Bijbehara (Anantnag) Kashmir, Anantnag, India; 3 University Centre for Research and Development, Chandigarh University, Mohali, India

**Keywords:** epidermal, frequency, grasses, morphotypes, silica

## Abstract

**Introduction:**

Taxonomic delimitation of closely related Echinochloa species is frequently complicated by morphological plasticity and overlapping vegetative characters, necessitating complementary diagnostic approaches. This study aimed to evaluate whether integrated phytolith characteristics can provide reliable discriminatory features for distinguishing Echinochloa colona and *E. crus-galli*.

**Methods:**

Phytolith biomineralization in *E. colona* and *E. crus-galli* was comparatively investigated by integrating epidermal silicification patterns, organ-specific phytolith morphology and occurrence, relative frequency, quantitative morphometry, silica accumulation, scanning electron microscopy (SEM), SEM–EDX elemental analysis, and multivariate statistics. Phytolith assemblages from roots, culms, leaves, and inflorescences were compared using presence–absence and morphometric data, while elemental composition was evaluated using principal component analysis.

**Results:**

Distinct phytolith distribution patterns occurred on the adaxial and abaxial leaf surfaces of the two species. Across the four organs, 50 phytolith morphotypes were recorded, of which 31 occurred in *E. colona* and 38 in *E. crus-galli*, with several showing species- or organ-restricted occurrence. Hierarchical cluster analysis demonstrated substantial differentiation among organspecific assemblages, with an overall Jaccard similarity of 0.229 between the species. Similarity was greatest between corresponding leaf and inflorescence assemblages (J = 0.292 each) and lowest between roots (J = 0.053). Silicified inflorescence ornamentation also differed, with ∩- and η-type patterns in *E. colona* and β-type patterns in *E. crus-galli*. Several shared morphotypes differed significantly in relative frequency, while quantitative morphometry revealed additional size and shape variation. Discriminant function analysis of 18 shared morphotype–organ combinations showed moderate morphometric differentiation (canonical correlation = 0.612), with an apparent classification accuracy of 83.3% and leave-one-pair-out cross-validated accuracy of 66.7%; phytolith width contributed most strongly to discrimination. SEM revealed additional ultrastructural variation, while SEM–EDX detected 14 elements in *E. colona* and 15 in *E. crus-galli*. Principal component analysis of elemental composition separated the two sampled species, with the first two components explaining 97.12% of the total variance.

**Discussion:**

The findings demonstrate that phytolith-based discrimination within Echinochloa is strengthened by integrating qualitative morphology, organ-specific occurrence, assemblage composition, relative frequency, morphometry, and elemental characteristics rather than relying on individual phytolith attributes. This integrative framework has potential applications in grass taxonomy, archaeobotany, and palaeoecological reconstruction, although broader populationlevel validation is required to establish the generality of the observed species-level differences.

## 1 Introduction

The genus *Echinochloa* P. Beauv. (Poaceae) comprises nearly 40–50 species of annual and perennial grasses distributed across tropical, subtropical, and temperate regions worldwide (Clayton et al., 2006; Michael, 2003; Hoste and Verloove, 2022; Wu et al., 2022). Species of the genus are ecologically successful because of their rapid growth, prolific seed production, broad environmental tolerance, and remarkable phenotypic plasticity. Among them, *Echinochloa crus-galli* (barnyard grass) and *E. colona* (jungle rice) are regarded as two of the world’s most aggressive agricultural weeds, infesting rice, maize, wheat, soybean and several other economically important crops (Holm et al., 1977; Michael, 2003; Yasuda et al., 2020). Their ability to thrive under contrasting moisture regimes, nutrient availability and climatic conditions, together with the evolution of herbicide resistance in many populations, has elevated these species to major global concerns for sustainable crop production (Heap, 2026). Morphological variability, interspecific resemblance and the occurrence of intermediate forms often complicate species identification, thereby necessitating the use of complementary taxonomic approaches that integrate anatomical, molecular and biomineralization-based characters (Yabuno, 1983; Shaheen et al., 2021; Wu et al., 2022; Naskar et al., 2024).

Like other grasses, *Echinochloa* species actively accumulate silicon from the soil and deposit it as hydrated amorphous silica within specialized cells and tissues during plant development. This process of biosilicification not only enhances structural rigidity and mechanical strength but also contributes to resistance against herbivory, pathogen attack, drought, salinity and other environmental stresses (Epstein, 1999; Ma and Yamaji, 2006; Hoskova et al., 2022). Following decomposition of plant tissues, these silica bodies persist in soils and sediments for thousands of years, thereby preserving a durable record of vegetation that is largely resistant to chemical and biological degradation (Piperno, 2006; Naskar et al., 2021; Biswas et al., 2024; Boyd et al., 2024). Because phytolith formation is under substantial genetic control while remaining responsive to environmental conditions, phytolith assemblages provide an important source of taxonomic, ecological and evolutionary information in grasses (Piperno, 2006; Naskar and Bera, 2018; Neumann et al., 2019; Morcote et al., 2025; Rudall et al., 2025). Consequently, phytolith analysis has become an increasingly valuable approach for distinguishing closely related grass taxa, reconstructing palaeovegetation, tracing crop–weed interactions and investigating the evolutionary ecology of economically important genera such as *Echinochloa* (Gallaher et al., 2020; Hoskova et al., 2022; Biswas et al., 2024).

Phytolith research in *Echinochloa* has developed from broad studies of grass silica bodies towards increasingly species-focused and archaeobotanical applications. Early work by Twiss et al. (1969) established a systematic framework for the classification of grass phytoliths and demonstrated their taxonomic significance within Poaceae, providing an important foundation for subsequent investigations of panicoid grasses. This framework was further developed by Piperno (1988, 2006), who emphasised the importance of modern reference collections for reliable phytolith identification and highlighted their wider applications in grass systematics, archaeobotany and palaeoenvironmental reconstruction. Archaeobotanical interest in *Echinochloa* was strengthened by Yang et al. (2015), who combined phytolith and starch-grain evidence from Early Neolithic contexts in the Lower Yangtze region and demonstrated the exploitation of barnyard grasses alongside rice more than 9,600 years ago, underscoring the importance of *Echinochloa* in early subsistence systems. Subsequently, Ge et al. (2018) undertook a detailed comparative investigation of phytoliths from the reproductive structures of nine *Echinochloa* species and identified morphological and morphometric characters with potential value for distinguishing barnyard millet and related taxa. Their use of quantitative measurements and discriminant analysis demonstrated the potential of phytolith characters for improving archaeobotanical identification within the genus. Later studies continued to explore *Echinochloa* phytoliths in archaeological and palaeoecological contexts while incorporating increasingly standardised nomenclature and quantitative approaches to improve taxonomic resolution (Ge et al., 2018). Despite these advances, most previous investigations have been directed primarily towards archaeological identification, whereas systematic comparisons of phytolith production among living *Echinochloa* species remain limited. In particular, integrated studies combining organ-specific morphology, quantitative morphometry, ultrastructural observations and elemental characterisation are still scarce. This gap provides a strong rationale for detailed comparative investigation of economically important and morphologically similar species such as *E. colona* and *E. crus-galli*.

Although phytolith morphology has been increasingly recognized as a useful character for grass identification, most studies have relied predominantly on qualitative descriptions of morphotypes, while quantitative morphometric characterization of closely related *Echinochloa* species remains comparatively limited. This is particularly important for *Echinochloa colona* and *Echinochloa crus-galli*, two closely related and morphologically similar grasses whose discrimination may be challenging, particularly when diagnostic macromorphological characters are incomplete or absent. Quantitative assessment of phytolith attributes such as size, shape and morphological proportions can provide objective and statistically testable characters, thereby complementing conventional qualitative morphotype identification (Ball et al., 2016). Indeed, morphometric approaches have been shown to improve the taxonomic resolution of phytolith analysis, particularly where closely related taxa produce broadly similar morphotypes (Ball et al., 2016). Recent geometric-morphometric studies have further demonstrated that quantitative analysis of phytolith shape can distinguish closely related grass species and can separate interspecific from intraspecific variation, highlighting the potential of phytolith morphometry for species-level discrimination (Hošková et al., 2021). Moreover, quantitative analyses of phytolith shape across diverse extant grasses have revealed significant phylogenetic and taxonomic signals, further supporting the value of morphometric approaches in grass systematics and palaeoecological reconstruction (Gallaher et al., 2020; Hošková et al., 2022). Establishing species-specific morphometric profiles for *E. colona* and *E. crus-galli* may therefore improve the discriminatory potential of phytoliths in *Echinochloa* and provide a modern reference dataset for interpreting phytolith assemblages recovered from soils, sediments and archaeological or palaeoecological deposits. Thus, quantitative phytolith morphometry represents an important complementary approach for assessing taxonomic relationships and expanding the utility of phytoliths in the identification and ecological interpretation of closely related grasses.

Despite the growing application of phytolith analysis in archaeobotany, systematic studies focusing on extant *Echinochloa* species remain extremely limited. To date, most investigations have concentrated on developing diagnostic criteria for identifying barnyard millet in archaeological assemblages, whereas comparative analyses of phytolith production among living *Echinochloa* species are scarce. Moreover, no previous study has integrated phytolith morphology with quantitative morphometry, frequency, SEM–EDX elemental characterization, and multivariate statistical analyses to evaluate species-level variation in *E. crus-galli* and *E. colona*. Consequently, the mechanisms underlying phytolith biomineralization, elemental composition and their taxonomic significance in these two globally important weed species remain poorly understood, providing the principal rationale for the present investigation.

## 2 Materials and methods

Specimens of *Echinochloa* (*E. colona & E. crus-galli*) were collected at the flowering stage from agricultural fields on the campus of Guru Nanak Dev University, Amritsar district, Punjab, India (32.64° N, 74.82° E) (**Figure 1**). One population of each species was collected for analysis. The specimens were identified using the online taxonomic resources GrassBase – The Online World Grass Flora (Clayton et al., 2006) and GrassWorld (Simon et al., 2014). Voucher specimens were prepared and deposited in the Herbarium of Guru Nanak Dev University, Amritsar, with voucher numbers 76147 for *E. colona* and 76148 for *E. crus-galli*. For phytolith analysis, the plants were separated into underground (root) and above-ground (culm, leaf and inflorescence) parts and preserved in 70% ethanol at 4 °C.

**Figure 1:**
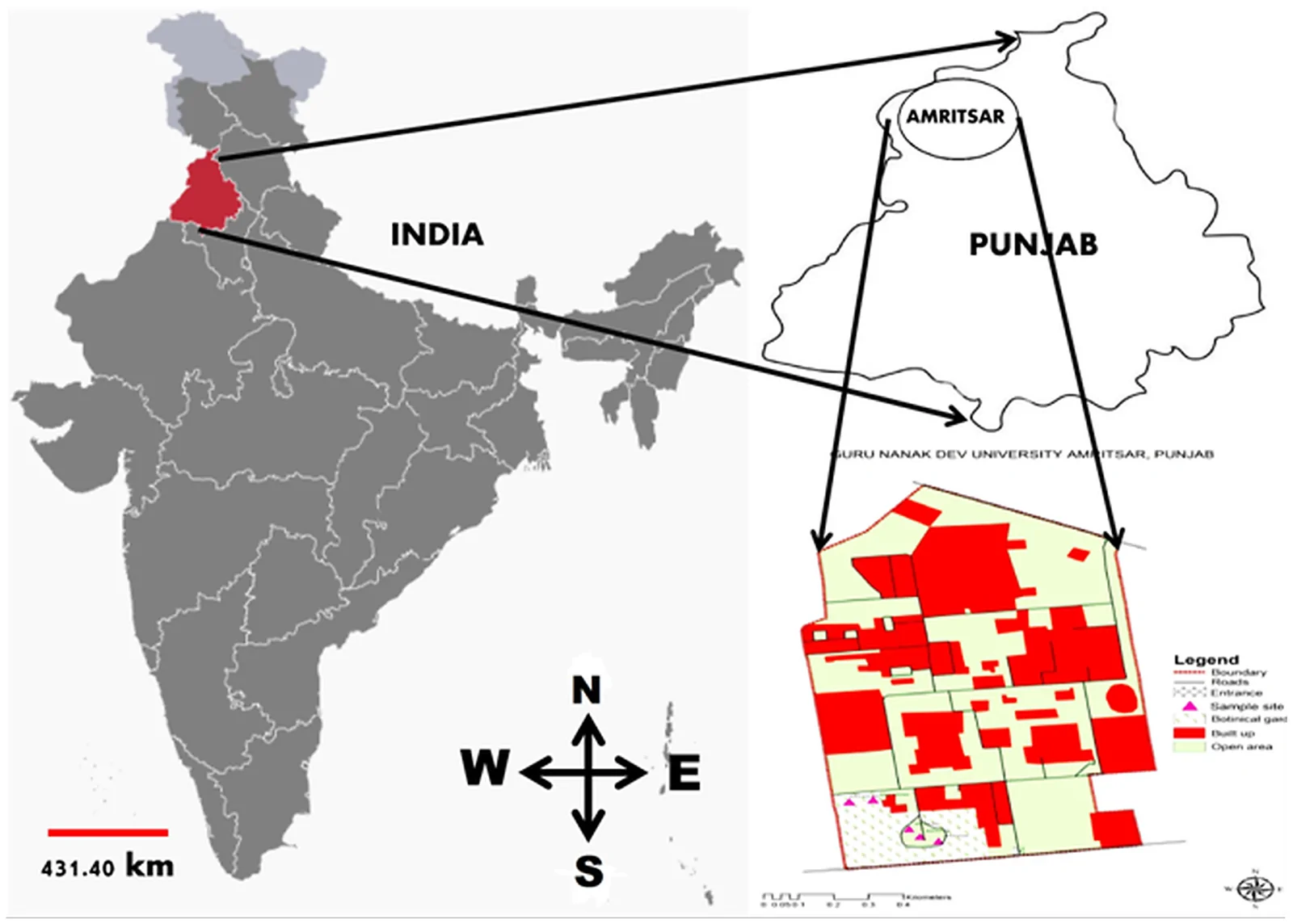
Map of sample collection site.

### 2.1 Phytolith analysis

#### 2.1.1 Epidermal *in situ* location

*In situ* localization and epidermal patterning of phytoliths on adaxial and abaxial leaf surfaces were examined following Clarke (1960) with minor modifications. Mature leaf segments were boiled in distilled water for 5–10 min, decolourized in ethanol at 80 °C, and further cleared in a lactic acid–chloral hydrate solution (3:1, v/v). Epidermal peelings from both leaf surfaces were obtained from the mid-region of the leaf blade, stained with gentian violet, dehydrated through a graded ethanol series, and mounted in DPX. Observations and microphotography were carried out using an Olympus binocular microscope equipped with a Micro Image Projection System (MIPS).

#### 2.1.2 Phytolith isolation

Phytoliths were isolated by dry ashing following Carnelli et al. (2001) with minor modifications. Approximately 40–50 plants of each species were separated into root, culm, leaf, and inflorescence, thoroughly washed, dried, weighed, and incinerated at 550 °C for 4–6 h. The resulting ash was treated sequentially with 30% H_2_O_2_ and 10% HCl at 80 °C, with repeated washing and centrifugation until a clear pellet was obtained. The final residue was dried at 60 °C. For microscopic observation, a small amount of dried ash was stained with gentian violet, mounted on slides, examined under an Olympus binocular microscope and photographed using MIPS at 40X magnification. Phytolith morphotypes from underground and aerial organs showed considerable morphological diversity and were named and classified following the International Code of Phytolith Nomenclature (ICPN 1.0 & 2.0) (Madella et al., 2005; Neumann et al., 2019).

### 2.2 Morphometry & frequency

Morphometric measurements of phytolith morphotypes were obtained using ImageJ software (v. 1.46r). Five size and shape descriptors were recorded following the recommendations of the International Committee for Phytolith Morphometrics (ICPM; Ball et al., 2016). The minimum adequate sample size (Nmin) was estimated using the formula Nmin = Z²α/2 × S²/(ME)², where Z²α/2 = 1.64 (two-tailed Z value at α = 0.10), S² is the variance, and ME represents 5% of the sample mean. This approach ensures 90% confidence that sample means fall within 5% of the population mean.

The relative frequency of individual phytolith morphotypes was estimated by counting 300 phytoliths on each slide. A total of five slides per organ (root, culm, leaf and inflorescence) were examined, resulting in the analysis of 1,500 phytoliths per organ.

### 2.3 Electron microscopy

Scanning electron microscopy (SEM) included mounting of isolated silica on aluminium stubs using double-sided adhesive tape, edge-coated with silver paint, dried at 40 °C, and sequentially coated with graphite and gold. Imaging was performed using a CARL ZEISS EVO 40 SEM operated at 40 kV.

### 2.4 Silica quantification

Quantification of silica from underground and above ground parts of *Echinochloa* species was carried out and percentage silica content (%) was calculated by the formula:


$$\text{Silica content }(\%)=\frac{\text{Final ash content }(\text{after digestion})}{\text{Dry mass of plant part}}\times 100$$

### 2.5 Elemental composition of phytoliths

Elemental composition of phytolith morphotypes was analyzed using Scanning Electron Microscopy coupled with Energy Dispersive X-ray analysis (SEM-EDX).

### 2.6 Statistical analysis

Descriptive statistical analyses of phytolith morphometric dimensions and elemental composition were performed using PAST (Paleontological Statistics) version 4.03 (Hammer et al., 2001). Discriminant function analysis (DFA) was first applied to phytolith morphometric data using morphotypes shared by both species within corresponding plant organs. Length, width, area and perimeter were used as predictor variables to assess morphometric discrimination between *Echinochloa colona* and *E. crus-galli*, and classification performance was evaluated using apparent classification accuracy and leave-one-pair-out cross-validation. Hierarchical cluster analysis was subsequently conducted on binary presence–absence data for 50 phytolith morphotypes recorded across roots, culms, leaves and inflorescences of both species. Jaccard dissimilarity was calculated among organ-specific assemblages, and clustering was performed using the average-linkage (UPGMA) method to assess similarities in phytolith assemblage composition between organs and species. Principal component analysis (PCA) was then applied to the elemental composition dataset to identify the principal sources of variation and to visualise differences in elemental profiles between *E. colona* and *E. crus-galli*.

## 3 Results

### 3.1 Foliar epidermal patterns

The present investigation revealed that both *Echinochloa colona* and *E. crus-galli* exhibited silica phytoliths predominantly localized in the costal regions of both leaf surfaces, whereas the intercostal regions remained largely devoid of silica deposition. However, interspecific differences were evident in the composition, distribution and organization of silica phytolith morphotypes (**Figures 2**, **3**).

**Figure 2:**
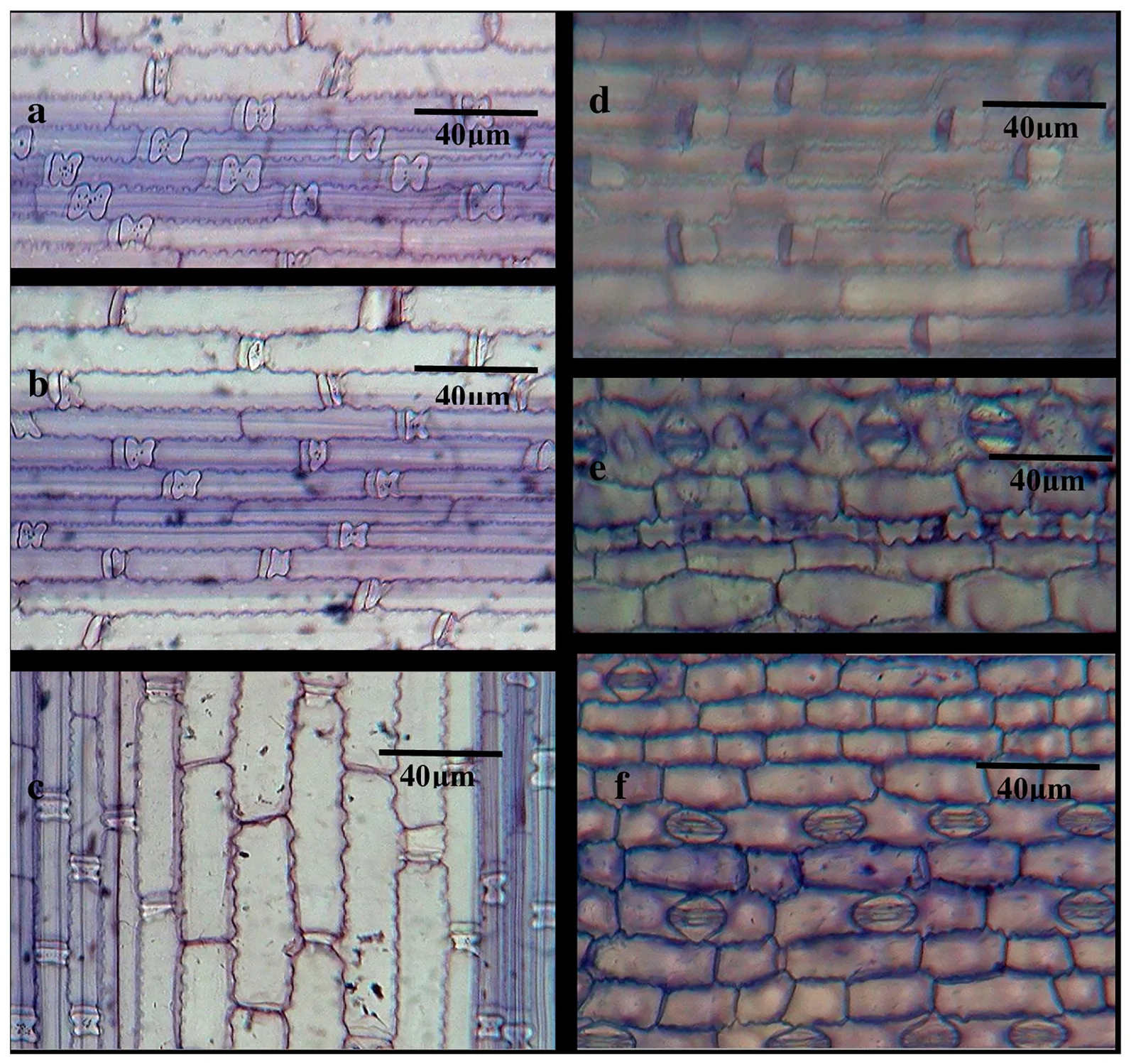
*In situ* location of foliar phytolith morphotypes of *Echinochloa colona* [Adaxial **(a–c)** and Abaxial **(d–f)** leaf surfaces].

**Figure 3:**
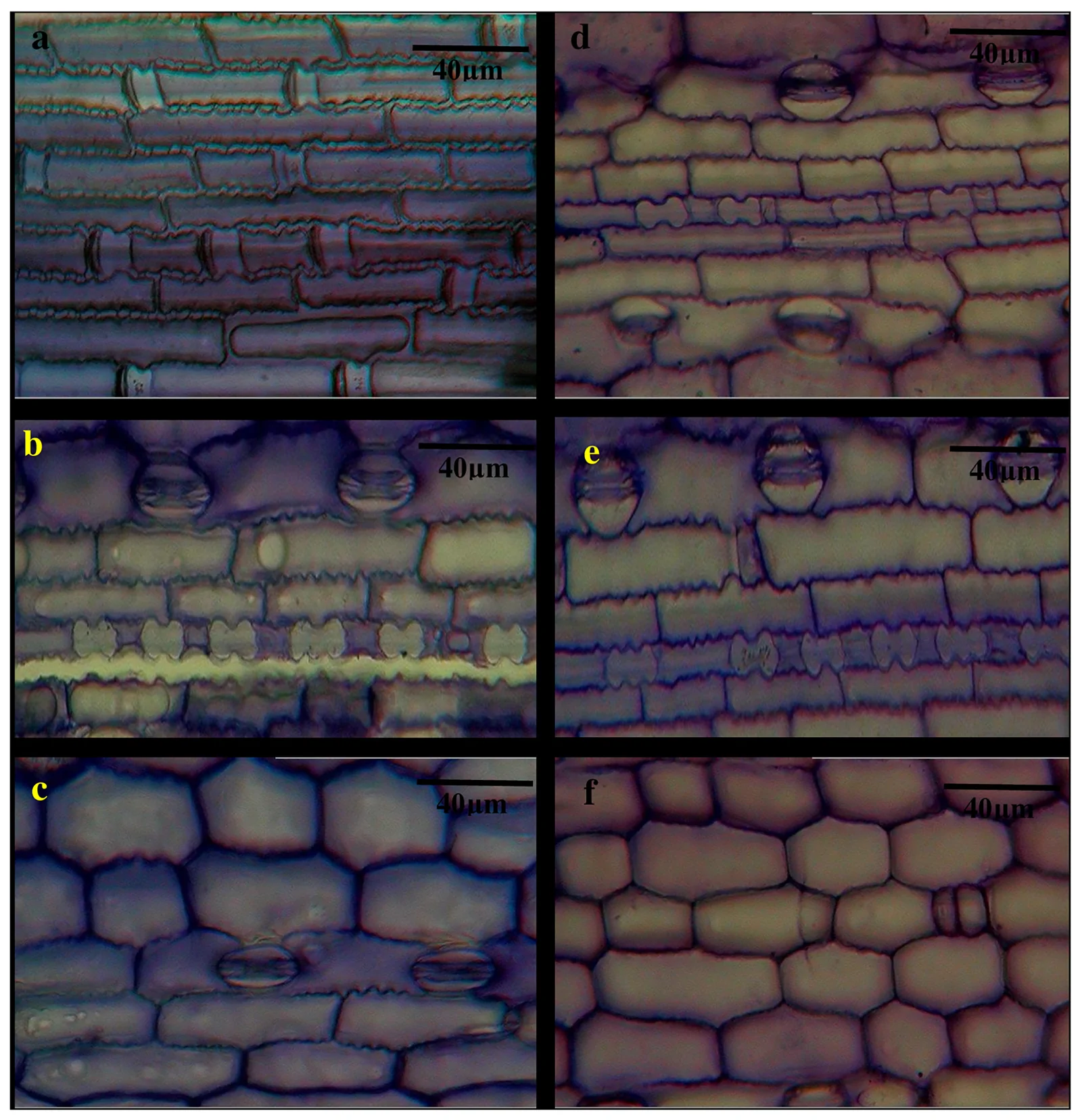
*In situ* location of foliar phytolith morphotypes of *Echinochloa crus-galli* [Adaxial **(a–c)** and Abaxial **(d–f)** leaf surfaces].

On the adaxial epidermis, *E. colona* was characterized by continuous files of bilobate silica cells possessing thick shanks and flat lobes, alternating regularly with epidermal long cells. Silica–cork cell pairs were conspicuous throughout the costal region (**Figures 2A–C**). In contrast, the adaxial epidermis of *E. crus-galli* exhibited greater morphological heterogeneity, with saddle phytoliths restricted to the midrib region, whereas bilobates with thick shanks and convex lobes, together with nodular bilobates, occurred along the lateral vascular bundles in association with cork cells. Flat-lobed bilobates were not observed in the analysed *E. crus-galli* material (**Figures 3A–C**).

Differences between the two species were also evident on the abaxial epidermis. In *E. colona*, the costal region consisted predominantly of abundant silica–cork cell pairs interspersed with files of bilobates bearing uneven lobes, nodular bilobates and polylobates, each consistently flanked by cork cells (**Figures 2D–F**). Stomata were confined to the intercostal region, where silica deposition was absent. Conversely, the abaxial epidermis of *E. crus-galli* comprised bilobates with thick shanks and convex lobes, nodular bilobates and comparatively fewer polylobates within the costal region, while the intercostal region lacked silica bodies (**Figures 3D–F**).

Overall, the two species differed in the composition and spatial distribution of silica-cell morphotypes. *E. crus-galli* showed saddle and convex-lobed bilobate morphotypes on the adaxial surface, whereas flat-lobed bilobates on the adaxial surface and uneven-lobed bilobates on the abaxial surface were observed in *E. colona*. The abundance and arrangement of silica–cork cell pairs also differed between the two species, particularly on the abaxial epidermis.

### 3.2 Ash and silica content

The present investigation revealed interspecific and inter-organ variation in ash and silica contents of *Echinochloa colona* and *E. crus-galli*. Ash content was highest in the culm of *E. crus-galli*, whereas the lowest ash contents were recorded in the leaves and inflorescences of *E. colona* (**Figure 4**).

**Figure 4:**
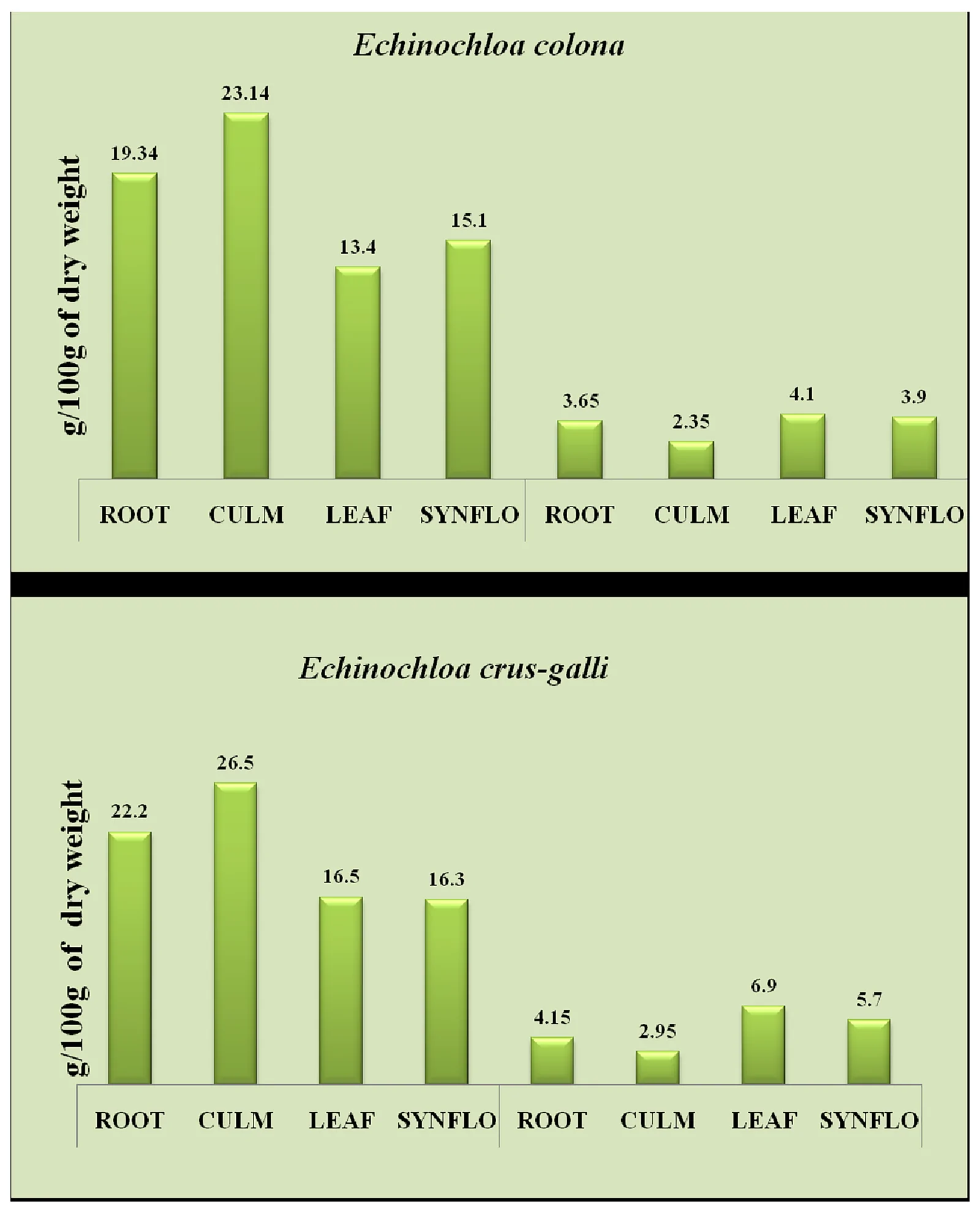
Ash and silica content in various plant parts of *E. colona* and *E. crus-galli*.

Silica accumulation showed a distinct organ-specific pattern in both species. Leaves contained the highest silica concentration, followed by inflorescences, roots and culms (**Figure 4**). Among the two species, *E. crus-galli* exhibited higher silica concentrations in both leaves and inflorescences than *E. colona*. In contrast, both species exhibited comparatively lower silica concentrations in roots and the least accumulation in culms.

### 3.3 Phytolith morphotypes

A total of 50 phytolith morphotypes were identified across *Echinochloa colona* and *E. crus-galli* (**Supplementary Table 1**). Of these, 31 morphotypes were recorded from *E. colona*, comprising 7 from roots, 8 from culms, 15 from leaves and 17 from inflorescences (**Figures 5A–D**). In comparison, *E. crus-galli* yielded 38 morphotypes, including 13 from roots, 12 from culms, 16 from leaves and 14 from inflorescences (**Figures 6A–D**).

**Figure 5:**
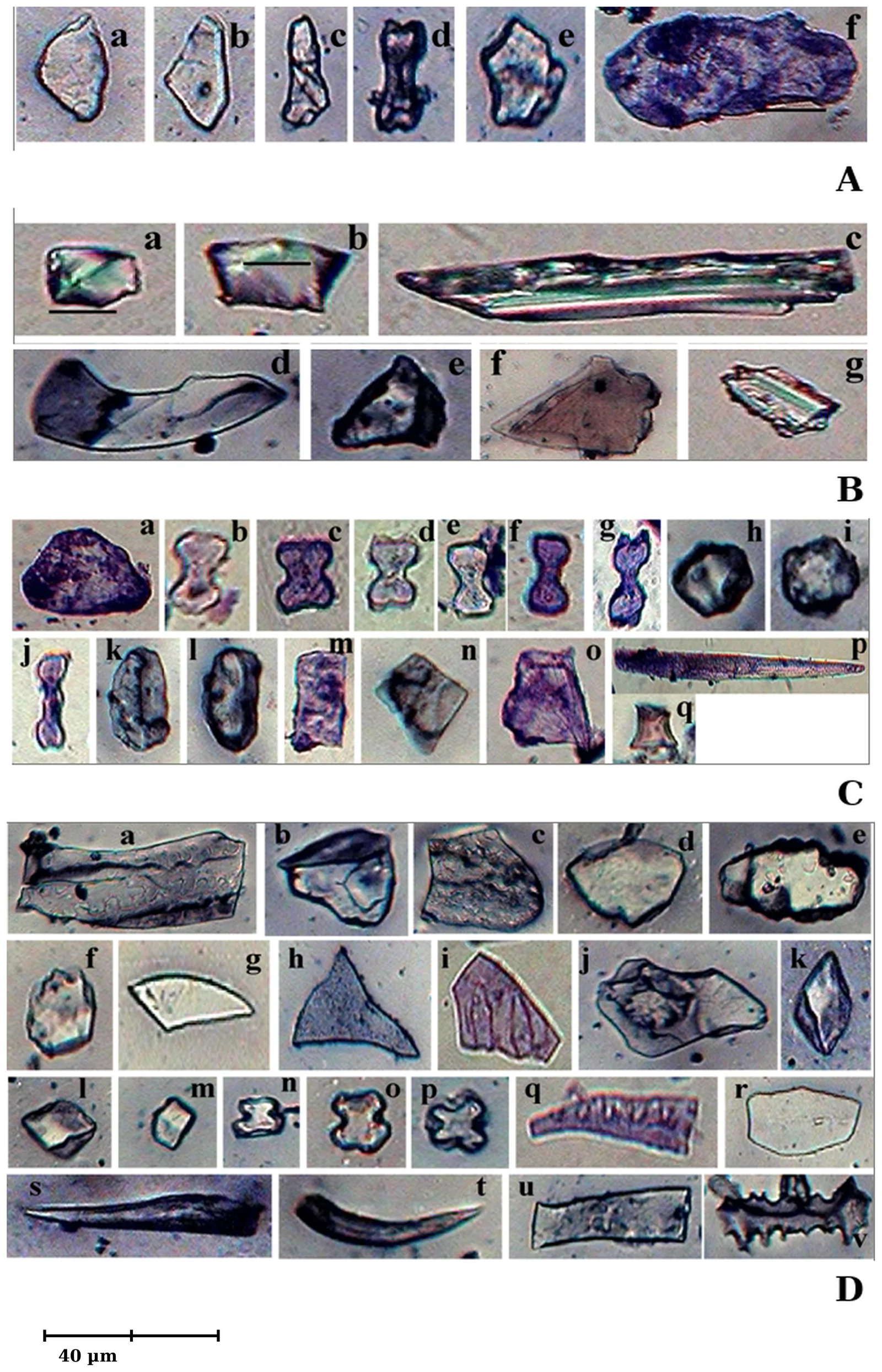
Phytolith morphotypes of *Echinochloa colona* **(A)** (root): oval **(a)**; scutiform **(b, c)**; bilobate with long shank and concave lobes **(d)**; trapezoid **(e)**; blocky irregular **(f)**; **(B)** (culm): cuboid **(a)**; rondel keeled **(b)**; elongate ridged **(c)**; scuntiform **(d)**; trapezoid **(e, f)**; parallepipedal **(g)**; **(C)** (leaf): spheroid irregular **(a)**; bilobates with short shank and convex lobes **(b)**; bilobates with short shank and flat lobes **(c, d)**; *Stipa* type bilobates **(e)**; bilobates with long shank and convex lobes **(f)**; bilobates with long shank and concave lobes **(g)**; spheroid polyhedral **(h)**; spheroid granulate **(i)**; nodular bilobate **(j)**; oval **(k, l)**; Parallepipedal **(m, n)**; cuneiform bulliform cell **(o)**; acicular **(p)**; tower flat **(q)**; **[D]** (inflorescence): blocky irregular **(a)**; trapezoid **(b, c)**; tabular polyhedral **(d)**; spheroid irregular **(e)**; oval **(f)**; scutiform **(g–j)**; fusiform **(k)**; cuboid **(l, m)**; bilobate with uneven lobes **(n)**; bilobate with short shank and concave lobes **(o–p)**; Clavate **(q)**; tabular Irregular **(r)**; macrohairs **(s)**; prickle hair **(t)**; rectangular **(u)**, dendritic **(v)**.

**Figure 6:**
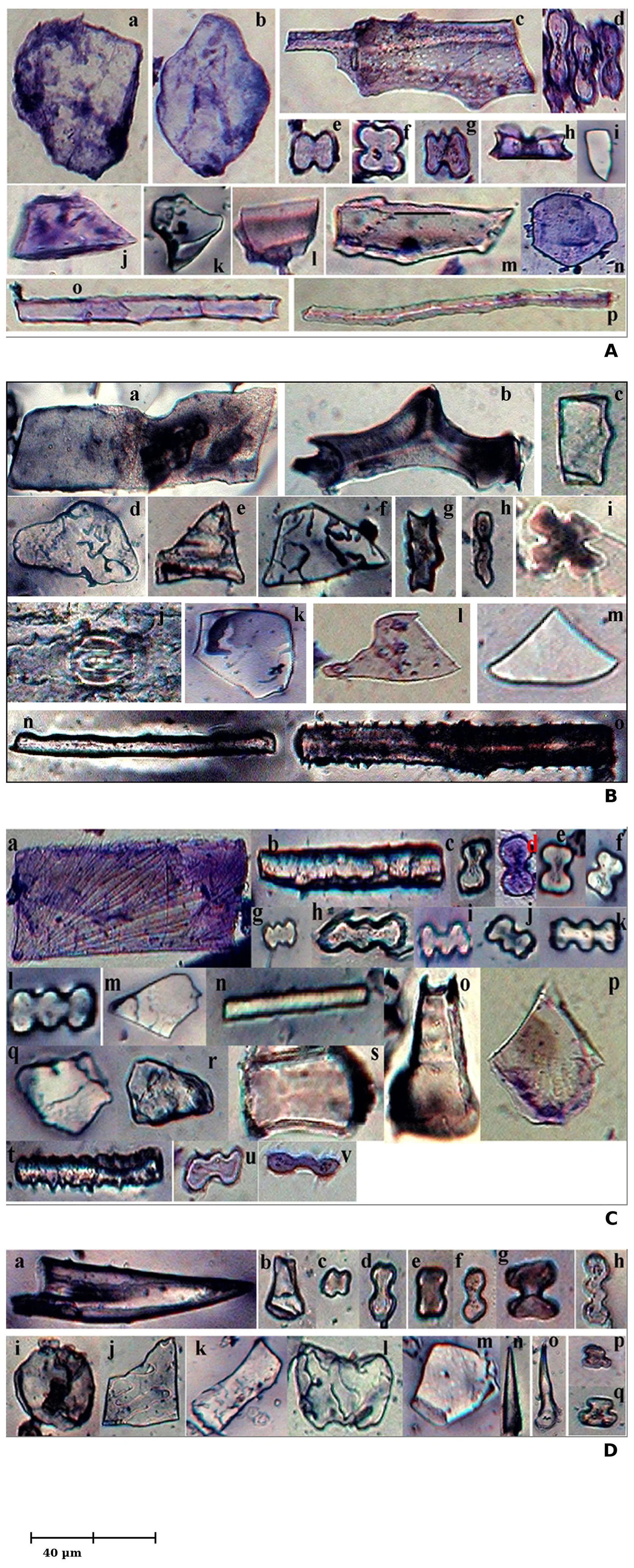
Phytolith morphotypes of Echinochloa crus-galli **(A)** (root): blocky polyhedral **(a)**; blocky irregular **(b)**; elongated tracheid **(c)**; Stipa type bilobates **(d)**; bilobate with short shank and flat lobes **(e)**; bilobates with short shank and concave lobes **(f)**; M-shaped nodular bilobate **(g)**; rondel (elongate–notched type) **(h)**; clavate **(i)**; tower flat **(j, k)**; parallepipedal **(l, m)**; orbicular **(n)**; elongate ridged **(o, p) (B)** (culm): tabular trachied **(a)**; trapezoid keeled trachied **(b)**; parallepipedal **(c)**; trapezoid **(d–f)**; saddle (narrow/deformed type) **(g)**; nodular bilobate **(h)**; cross **(i)**; stomata **(j)**; Tabular polyhedral **(k)**; Scuntiform **(l, m)**; Elongate smooth **(n)**; Elongate dentate **(o) (C)** (Leaf): Rectangular **(a)**; Cylindrical **(b)**; Stipa type bilobate **(c)** Bilobate with short shank and convex lobes **(d)**; Bilobates with short shank and flat lobes **(e, f)**; Polylobate irregular **(g, h)**; M-shaped nodular bilobates **(i, j)**; Polylobate **(k, l)**; Scutiform **(m)**; Elongate smooth **(n)**; Cuneiform bulliform cell **(o)**; Parallepipedal bulliform cell **(p)**; Trapezoid **(q, r)**; parallepipedal **(s)** Elongate dentate **(t)** Nodular bilobate **(u, v) (D)** (Inflorescence): Macrohair **(a)**; Tower flat **(b)**; Saddle **(c)**; Bilobate with long shank and concave lobes **(d)**; Stipa type bilobates **(e, f)** Bilobate with short shank and flat lobes **(g)**; Nodular bilobate **(h)**: Spheroid irregular **(i)**; Scutiform **(j)**; Parallepipedal **(k)**; Blocky irregular **(l)**; Tabular polyhedral **(m)**; Prickle **(n, o)**; Bilobate with uneven lobes **(p, q)**.

The phytolith assemblages differed among organs and between the two species. Both underground and aboveground organs contributed to the total phytolith diversity, with individual organs containing distinct combinations of morphotypes.

The diagnostic significance of the identified morphotypes was evaluated using ubiquity values and the diagnostic index (**Supplementary Table 1**). Morphotypes restricted to one of the two species exhibited a ubiquity value and diagnostic index of 0.50, whereas morphotypes restricted to a particular organ showed a ubiquity value of 0.125. A ubiquity value of 1.0 indicated that a morphotype was shared among corresponding organs of both species. Morphotypes with ubiquity values exceeding 0.125 occurred in more than one organ and showed wider organ-level distribution.

Several morphotypes were recorded exclusively in *E. colona*, including acicular, bilobate with long shank and convex lobes, cuboid, dendritic, fusiform, rectangular, rectangular with columellate extensions, rondel keeled, spheroid granulate, spheroid polyhedral and tabular irregular phytoliths.

In contrast, the analysed *E. crus-galli* material showed the exclusive occurrence of blocky polyhedral, blocky rectangular, cross, cylindrical, elongate dendritic, elongate smooth, tracheid, M-shaped nodular bilobate, orbicular, parallelepipedal bulliform cell, polylobate irregular, rondel (elongate notched type), rondel complex, saddle, saddle (narrow/deformed type), tabular tracheid and trapezoid keeled tracheid phytoliths.

The organ-wise distribution and complete occurrence pattern of all 50 phytolith morphotypes are presented in **Supplementary Table 1** and **Figures 5**, **6**.

SEM examination further revealed variation in the ultrastructural characteristics of several phytolith morphotypes (**Figure 7**). Spheroid phytoliths were differentiated into spheroid granulate, spheroid irregular and spheroid polyhedral forms on the basis of differences in their surface characteristics and overall morphology (**Figures 7A, B**).

**Figure 7:**
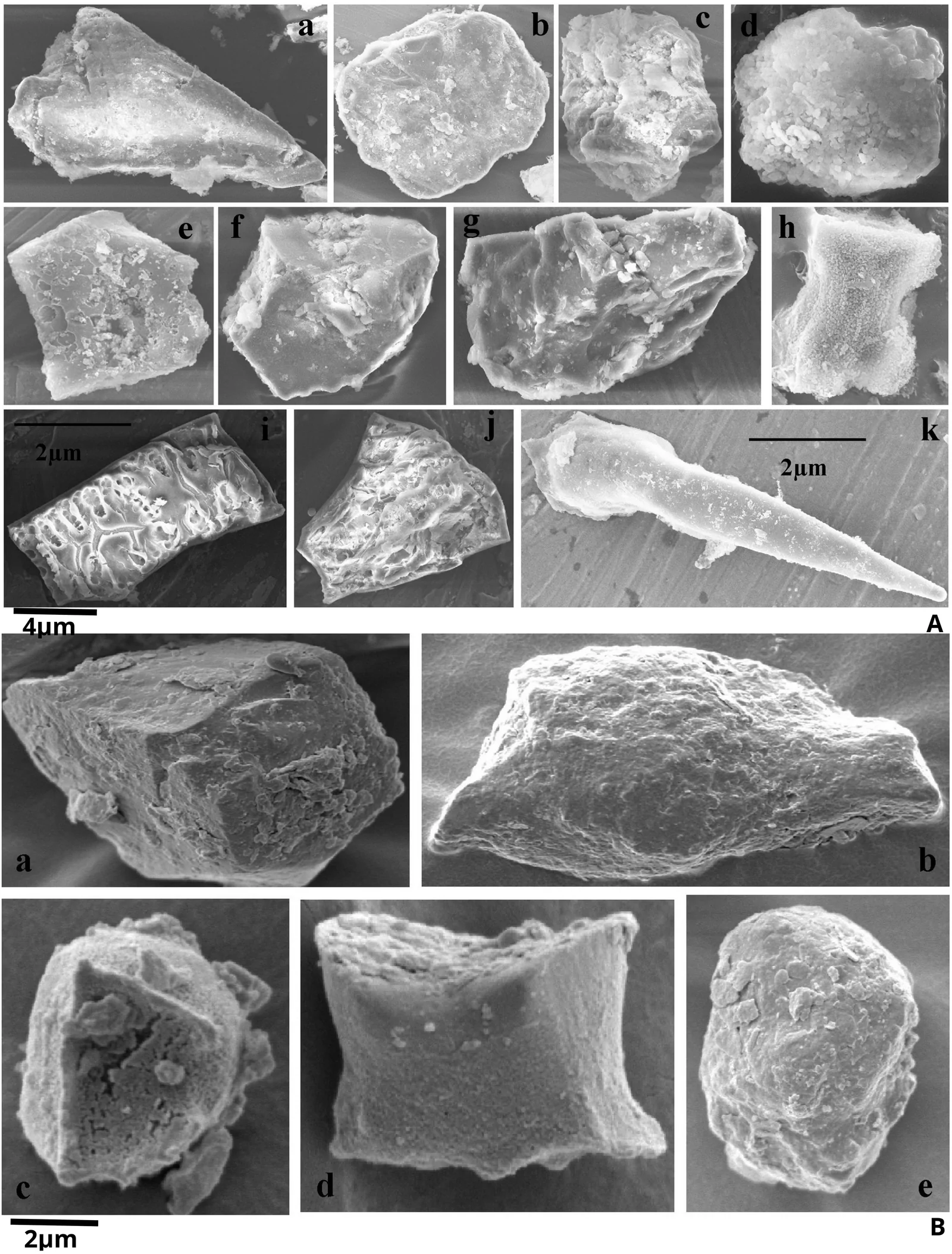
Scanning electron micrographs of phytolith morphotypes of **(A)** *Echinochloa colona* (root): scutiform **(a)**; spheroid irregular **(b)** spheroid granulate **(c, d)** (culm): tabular polyhedral **(e)**; spheroid polyhedral **(f)** (leaf): trapezoid **(g)**; *Stipa* type bilobate **(h)** inflorescence: rectangular with columellate extensions **(i)**; trapezoid **(j)**; macrohairs **(k)**. **(B)** *Echinochloa crus-galli* (inflorescence): Spheroid polyhedral **(a)**; Trapezoid **(b)**; Spheroid irregular **(c)**; Rondel complex **(d)**; Tabular irregular **(e)**.

Similarly, rondel phytoliths were differentiated into rondel complex, rondel keeled and rondel (elongate notched type) based on differences in keel development, marginal configuration and overall morphology (**Figure 7B**).

The detailed morphological characteristics and organ-wise distribution of the identified phytolith morphotypes are summarized in **Supplementary Table 1** and illustrated in **Figures 5**–**7**.

Hierarchical cluster analysis revealed considerable differentiation in the organ-specific phytolith assemblages of *Echinochloa colona* and *E. crus-galli* (**Figure 8**). Based on 50 morphotypes scored separately across root, culm, leaf and inflorescence, the overall Jaccard similarity coefficient was 0.229, corresponding to a Jaccard dissimilarity of 0.771. Similarity was highest for leaf and inflorescence assemblages (J = 0.292 each), followed by culm (J = 0.250), whereas root phytolith assemblages showed very low similarity (J = 0.053). Hierarchical clustering based on Jaccard distance and average linkage further reflected the differentiation of the two species, with leaf and inflorescence assemblages showing the closest cross-species affinities.

**Figure 8:**
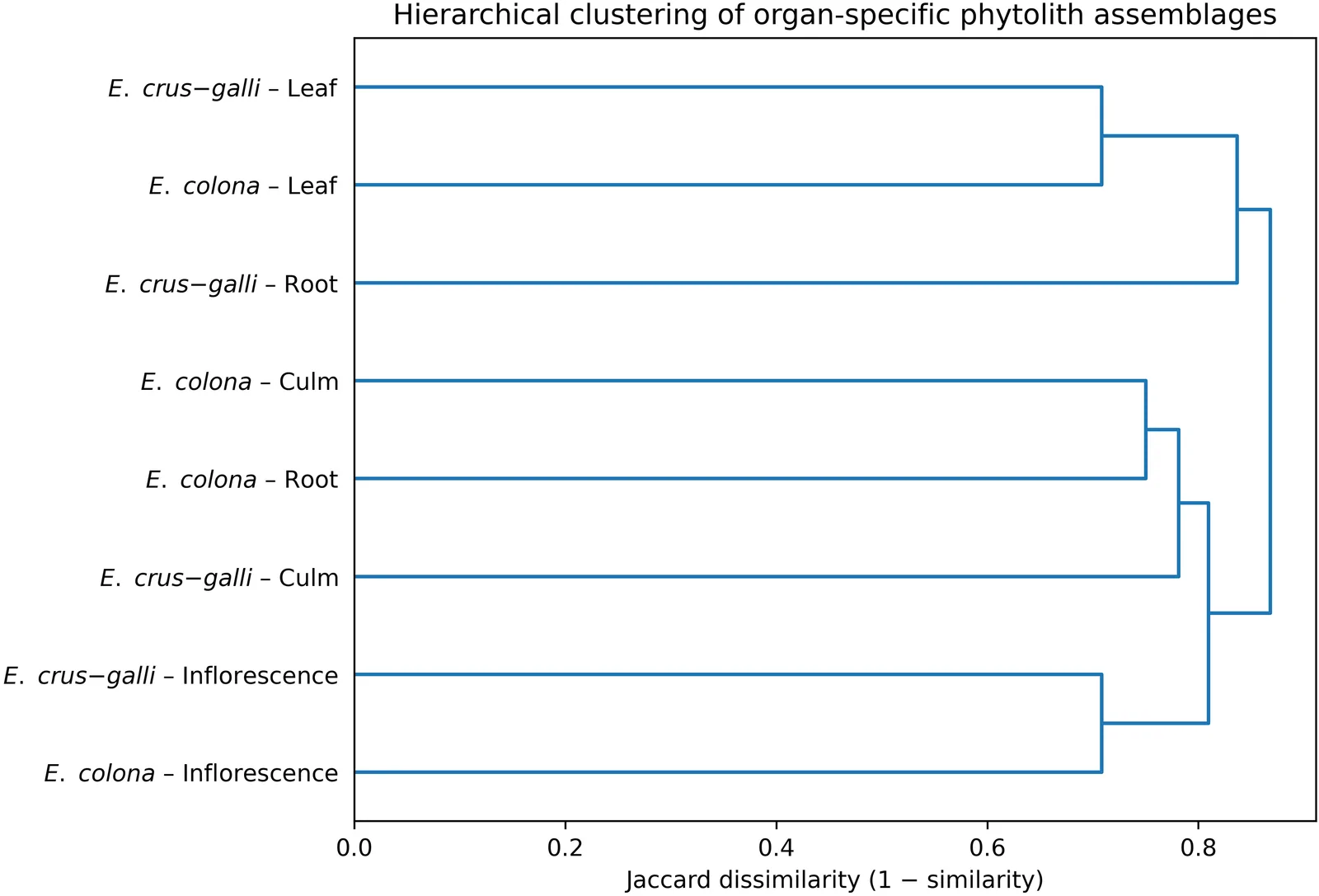
Hierarchical clustering of organ-specific phytolith assemblages of *Echinochloa colona* and *E. crus-galli* based on presence–absence data for 50 phytolith morphotypes using Jaccard dissimilarity.

### 3.4 Inflorescence epidermal ornamentation and undulation patterns

The investigated *Echinochloa* species exhibited differences in the silicified undulated ornamentation patterns of the inflorescence bracts. These ornamentation patterns observed on the phytolith surfaces were categorized according to their characteristic morphological configurations as ∩-type (inverted U-shaped), Ω-type (omega-shaped), η-type (eta-shaped), β-type (beta-shaped) and Λ-type (lambda-shaped). These symbol-based categories are used consistently throughout the manuscript and are illustrated in **Figure 9**.

**Figure 9:**
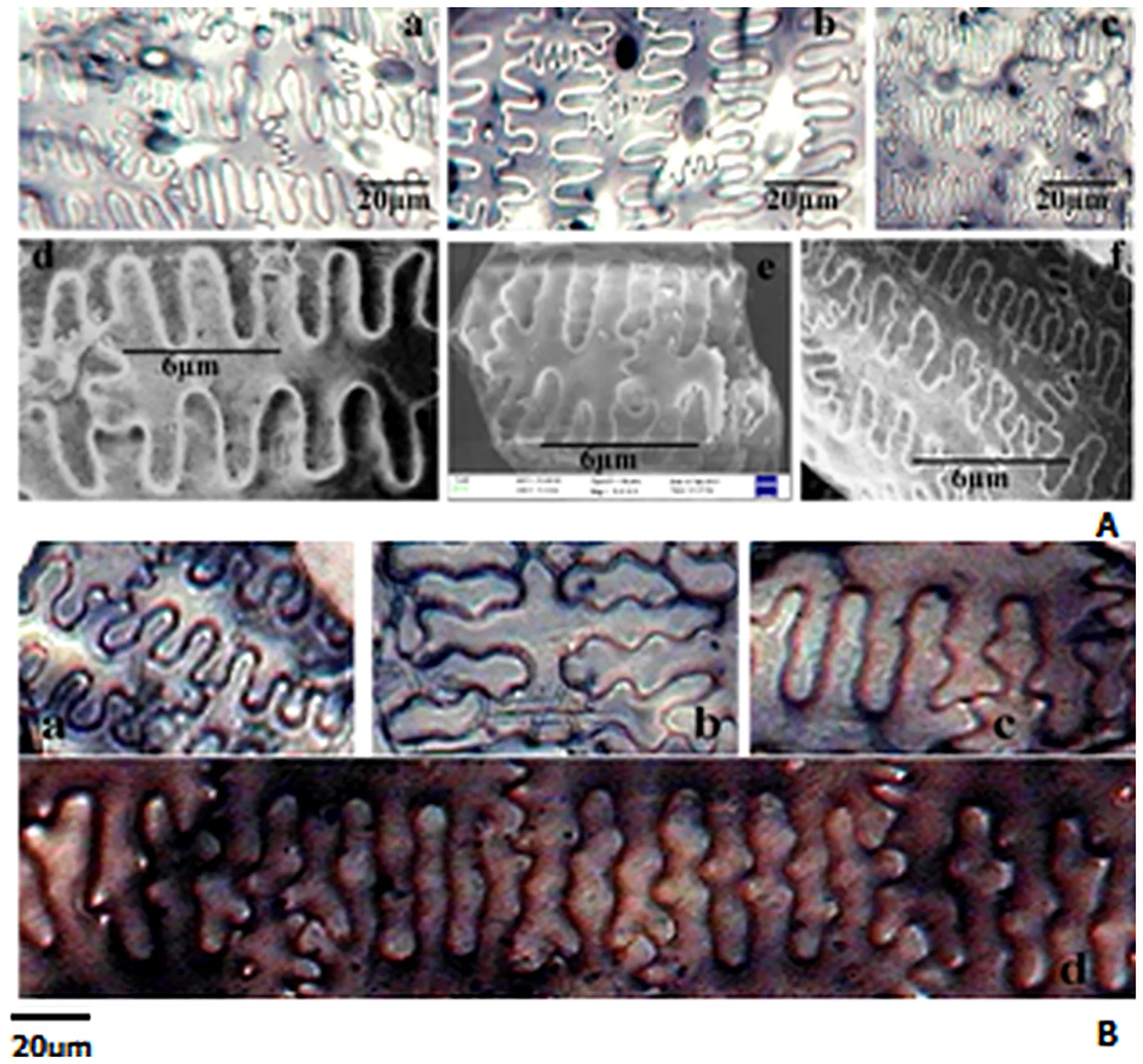
Undulated patterns and ornamentations on the epidermal long cells of *Echinochloa* inflorescence bracts/glumes (lemma & palea). **(A)** *Echinochloa colona*- ∩ and η-type **(a, b, d)**; η-I & η-II type **(c, e, f) (B)** *Echinochloa crus-galli*-β-I & β-II type **(a)**; β-II & β-III type **(b, c)**; β-IV type **(d)** of epidermal undulation patterns.

*E. colona* showed ∩-type and η-type undulated ornamentations (**Figures 9A-a–B, D**). The η-type was further differentiated into η-I and η-II subtypes based on differences in the amplitude of epidermal cell undulations (**Figures 9A-c, E, F**).

In contrast, *E. crus-galli* exhibited β-type undulated ornamentations, represented by β-I and β-II subtypes (**Figures 9B-a**), β-II and β-III subtypes (**Figures 9B-b–C**), and β-IV subtype (**Figures 9B-d**). ∩- and η-type ornamentations were not observed in the analysed *E. crus-galli* material, whereas β-type ornamentation was not recorded in the analyzed *E. colona* material.

### 3.5 Frequency distributions and morphometric measurements

The relative frequencies of several shared phytolith morphotypes differed between *E. colona* and *E. crus-galli* (**Figure 10**). For example, prickle hair phytoliths occurring in the inflorescences of both species exhibited an average relative frequency of 9% in *E. colona* and 4% in *E. crus-galli* (*P* ≤ 0.05; χ² test). Similarly, trapezoid phytoliths isolated from the culms accounted for an average frequency of 15% in *E. colona* and 7% in *E. crus-galli* (*P* ≤ 0.05; χ² test).

**Figure 10:**
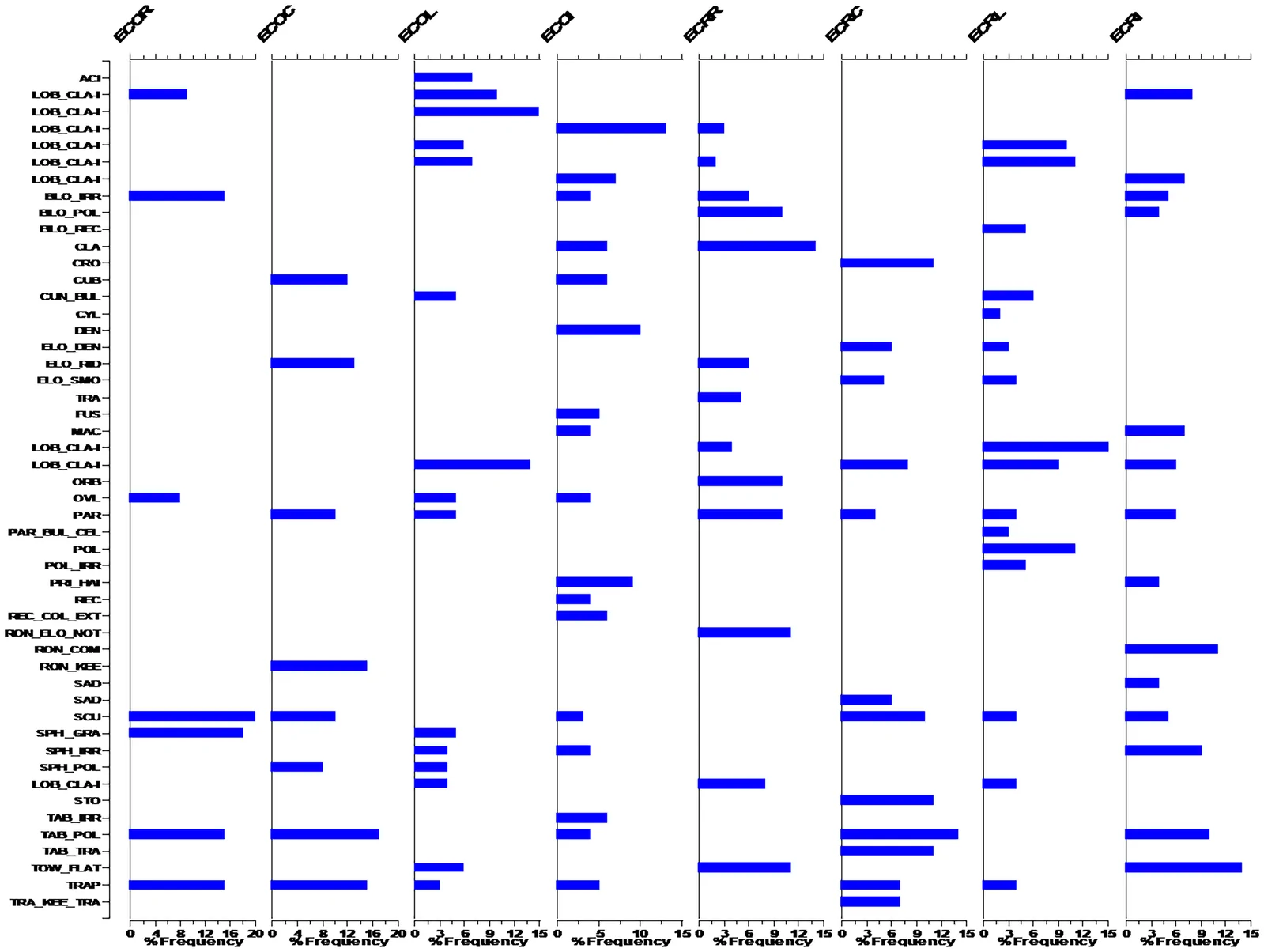
Stratigraphic diagram showing percentage frequency of different phytolith morphotypes (for description of phytolith morphotypes, refer to **Supplementary Table 1**) (ECOR, *Echinochloa colona* Root; ECOC, *Echinochloa colona* Culm;ECOL, *Echinochloa colona* Leaf; ECOI, *Echinochloa colona* Inflorescence; ECRR, *Echinochloa crus-galli* Root; ECRC, *Echinochloa crus-galli* Culm; ECRL, *Echinochloa crus-galli* Leaf; ECRI, *Echinochloa crus-galli* Inflorescence.

The morphometric measurements of the analysed phytolith morphotypes are presented in **Supplementary Tables 2**, **3**. Differences between the two species were observed in the measured quantitative attributes, including length, width, area, perimeter and aspect ratio.

Discriminant function analysis of 18 shared morphotype–organ combinations revealed significant morphometric separation between *Echinochloa colona* and *E. crus-galli* (**Figure 11**). The single discriminant function had an eigenvalue of 0.599 and a canonical correlation of 0.612 (Wilks’ λ = 0.626). Apparent classification accuracy was 83.3%, while leave-one-pair-out cross-validation yielded an accuracy of 66.7%. Among the morphometric variables, width contributed most strongly to discrimination, followed by area and perimeter, whereas length showed a comparatively weaker contribution. The greatest separation was observed for inflorescence macrohair and scutiform phytoliths, leaf bilobates with short shank and flat lobes, leaf *Stipa*-type bilobates, culm trapezoids and scutiform phytoliths, leaf nodular bilobates, and inflorescence bilobates with uneven lobes.

**Figure 11:**
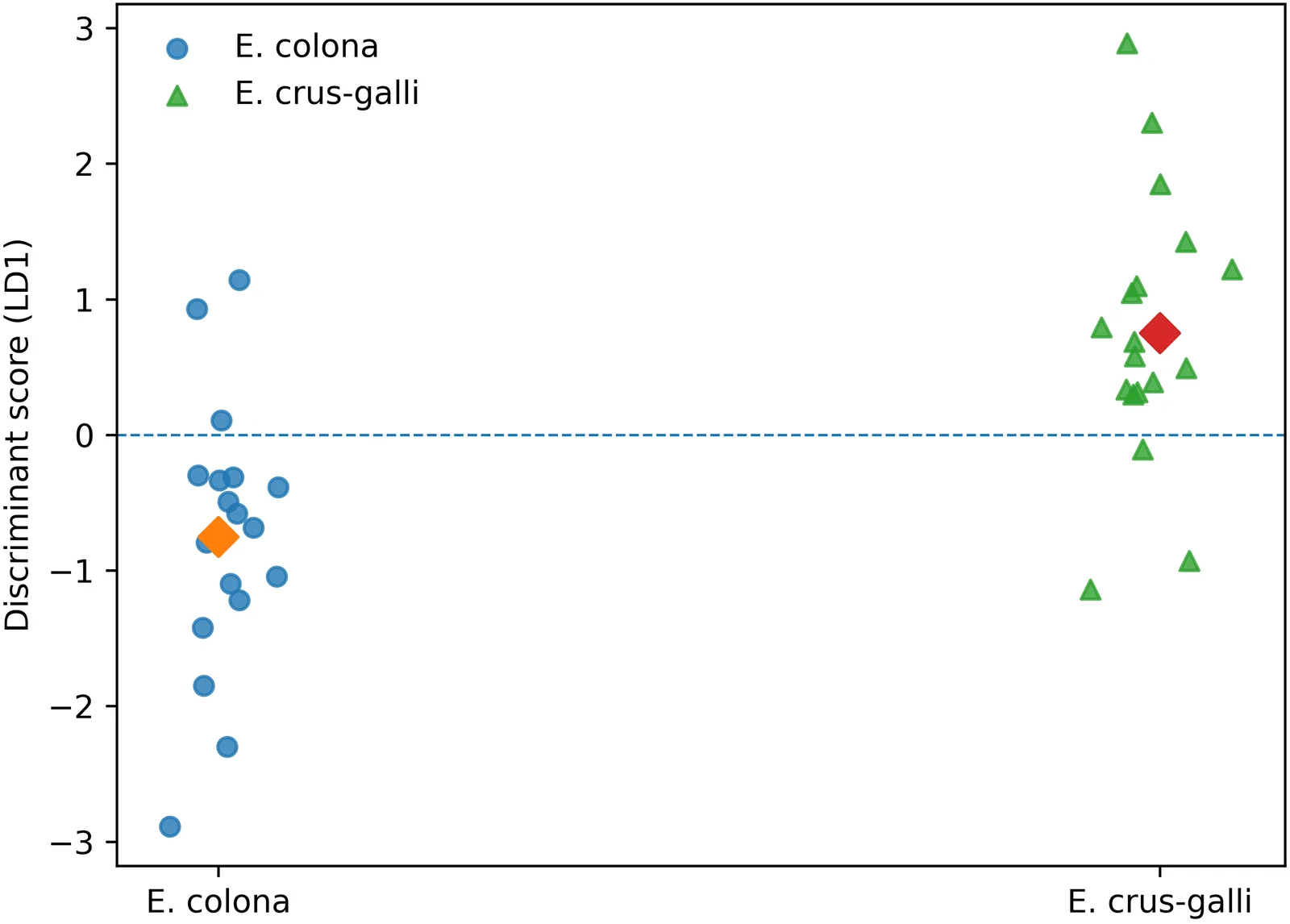
Discriminant function analysis (DFA) of 18 shared phytolith morphotype–organ combinations of *Echinochloa colona* and *E. crus-galli* based on length, width, area and perimeter measurements.

### 3.6 Anatomical origin of phytolith morphotypes

The anatomical origin of the isolated phytolith morphotypes was established by correlating their morphology with the corresponding silica-producing cell types in different plant organs (**Table 1**). The observed phytolith morphotypes were associated with specific cell and tissue types within the examined roots, culms, leaves and inflorescences. The anatomical origin and corresponding tissue associations of the identified morphotypes are summarized in **Table 1**.

**Table 1:** Anatomical origin of various phytolith morphotypes reported in present study.

S.NO	Phytolith Morphotype	Anatomical Origin	References(s)
01.	Acicular	Epidermal trichomes, sclerenchyma	Metcalfe,1960; Piperno, 2006
02.	Bilobate (long shank, concave lobes)	Foliar epidermal short cells	Twiss et al., 1969; Strömberg, 2004
03.	Bilobate (long shank,convex lobes)	Foliar epidermal short cells	Twiss et al., 1969; Piperno, 2006
04.	Bilobate (short shank,concave lobes)	Foliar epidermal short cells	Twiss et al., 1969; Strömberg, 2011
05.	Bilobate (short shank,convex lobes)	Foliar epidermal short cells	Twiss et al., 1969; Madella et al., 2005
06.	Bilobate (short shank,flat lobes)	Foliar epidermal short cells	Piperno, 2006; Strömberg, 2004
07.	Bilobate (uneven lobes)	Foliar epidermal short cells	Madella et al., 2005
08.	Blocky irregular	Mesophyll, sclerenchyma	Piperno, 2006; Hodson et al., 2005
09.	Blocky polyhedral	Parenchyma,vascular tissue	Metcalfe,1960; Piperno, 2006
10.	Blocky rectangular	Bulliform (motor) cells, epidermal tissue	Bhat et al., 2018; Twiss et al., 1969
11.	Clavate	Trichomes, hairs	Metcalfe,1960; Prat, 1936
12.	Cross	Foliar epidermal short cells	Twiss et al., 1969; Piperno, 2006
13.	Cuboid	Parenchyma, sclerenchyma	Hodson et al., 2005
14.	Cuneiform bulliform cell	Bulliform (motor) cells	Metcalfe,1960; Piperno, 2006
15.	Cylindrical	Fibers, tracheary tissue	Prat, 1936; Metcalfe,1960
16.	Dendritic	Epidermal cells of Inflorescence bracts, lemma/palea	Twiss et al., 1969; Piperno, 2006
17.	Elongate dendritic	Long epidermal cells	Metcalfe,1960; Madella et al., 2005
18.	Elongate ridged	Long epidermal cells	Prat, 1936; Piperno, 2006
19.	Elongate smooth	Long epidermal cells	Metcalfe,1960
20.	Elongate tracheid	Tracheary elements	Metcalfe,1960; Solomonova, 2025
21.	Fusiform	Epidermal long cells or specialized mesophyll cells	Mulholland & Rapp, 1992
22.	Macrohairs	Trichomes	Metcalfe,1960
23.	M-shaped nodular bilobate	Epidermal short cells	Twiss et al., 1969; Strömberg, 2011
24.	Nodular bilobate	Foliar epidermal short cells	Twiss et al., 1969
25.	Orbicular	Parenchyma	Piperno, 2006
26.	Oval	Parenchyma	Hodson et al., 2005
27.	Parallelepipedal	Parenchyma, sclerenchyma	Metcalfe,1960

### 3.7 Elemental composition of phytoliths

SEM–EDX analysis of phytolith preparations isolated from different vegetative and reproductive parts of the two *Echinochloa* species revealed elemental variation (**Table 2**; **Figures 12**, **13**). Fourteen elements were detected in *E. colona*: aluminium (Al), carbon (C), calcium (Ca), chlorine (Cl), copper (Cu), iron (Fe), potassium (K), magnesium (Mg), manganese (Mn), sodium (Na), oxygen (O), rubidium (Rb), silicon (Si) and strontium (Sr). Fifteen elements were detected in *E. crus-galli*: aluminium (Al), carbon (C), calcium (Ca), chlorine (Cl), copper (Cu), iron (Fe), potassium (K), magnesium (Mg), nitrogen (N), sodium (Na), oxygen (O), sulphur (S), silicon (Si), strontium (Sr) and titanium (Ti).

**Table 2:** Elemental composition of phytolith morphotypes from various parts of *Echinochloa* spp.

Species	*Echinochloa colona*								*Echinochloa crus-galli*							
Plant part	Root		Culm		Leaf		Inflorescence		Root		Culm		Leaf		Inflorescence	
Element	WT%	AT%	WT%	AT%	WT%	AT%	WT%	AT%	WT%	AT%	WT%	AT%	WT%	AT%	WT%	AT%
**Al**	**8.56**	**6.872**	**--------**	**--------**	**5.43**	**4.482**	**--------**	**--------**	**1.342**	**0.866**	**5.058**	**3.47**	**2.884**	**2.13**	**2.374**	**1.552**
**C**	**4.28**	**6.776**	**4.93**	**8.944**	**--------**	**--------**	**--------**	**--------**	**34.196**	**44.028**	**24.38**	**35.222**	**28.838**	**38.348**	**35.024**	**46.988**
**Ca**	**0.512**	**0.242**	**--------**	**--------**	**--------**	**--------**	**--------**	**--------**	**0.138**	**0.048**	**1.124**	**0.542**	**0.36**	**0.146**	**0.778**	**0.356**
**Cl**	**--------**	**--------**	**0.578**	**0.356**	**--------**	**--------**	**--------**	**--------**	**1.182**	**0.508**	**0.514**	**0.258**	**1.314**	**0.626**	**0.464**	**0.212**
**Cu**	**--------**	**--------**	**5.57**	**2.048**	**2.586**	**0.88**	**2.1**	**0.698**	**0.24**	**0.064**	**0.372**	**0.104**	**0.434**	**0.116**	**0.324**	**0.088**
**Fe**	**4.45067**	**4.63**	**3.69267**	**3.78267**	**0.276**	**0.112**	**--------**	**--------**	**--------**	**--------**	**1.966**	**0.654**	**0.306**	**0.108**	**--------**	**--------**
**K**	**--------**	**--------**	**--------**	**--------**	**2.088**	**1.208**	**1.206**	**0.648**	**0.218**	**0.082**	**1.046**	**0.482**	**1.952**	**0.994**	**1.944**	**0.924**
**Mg**	**2.48133**	**2.436**	**--------**	**--------**	**--------**	**--------**	**--------**	**--------**	**0.228**	**0.154**	**0.62**	**0.472**	**0.222**	**0.174**	**0.13**	**0.096**
**Mn**	**3.466**	**3.533**	**--------**	**--------**	**--------**	**--------**	**--------**	**--------**	**--------**	**--------**	**--------**	**--------**	**--------**	**--------**	**--------**	**--------**
**N**	**--------**	**--------**	**--------**	**--------**	**--------**	**--------**	**--------**	**--------**	**--------**	**--------**	**--------**	**--------**	**4.062**	**3.894**	**--------**	**--------**
**Na**	**--------**	**--------**	**--------**	**--------**	**1.19**	**1.096**	**--------**	**--------**	**1.1**	**0.76**	**0.966**	**0.762**	**0.072**	**0.062**	**0.5**	**0.358**
**O**	**43.224**	**58.052**	**46.198**	**65.756**	**41.386**	**55.972**	**45.876**	**60.468**	**37.36**	**38.828**	**38.428**	**42.068**	**34.734**	**37.612**	**31.832**	**33.154**
**Rb**	**--------**	**--------**	**8.136**	**2.78**	**--------**	**--------**	**--------**	**--------**	**--------**	**--------**	**--------**	**--------**	**--------**	**--------**	**--------**	**--------**
**S**	**--------**	**--------**	**--------**	**--------**	**--------**	**--------**	**--------**	**--------**	**0.156**	**0.066**	**--------**	**--------**	**--------**	**--------**	**0.062**	**0.028**
**Si**	**38.21**	**29.438**	**37.764**	**28.65**	**47.042**	**36.248**	**50.82**	**38.186**	**23.844**	**14.6**	**25.464**	**15.94**	**24.722**	**15.75**	**26.568**	**16.24**
**Sr**	**--------**	**--------**	**1.738**	**0.578**	**--------**	**--------**	**--------**	**--------**	**--------**	**--------**	**0.06**	**0.022**			**--------**	**--------**
**Ti**	**--------**	**--------**	**--------**	**--------**	**--------**	**--------**	**--------**	**--------**	**--------**	**--------**	**--------**	**--------**	**0.102**	**0.038**	**--------**	**--------**

Entries in the Table indicate Mean±Standard error; (--------), Absence of element; WT%, Weight percentage and AT%, Atomic percentage.

**Figure 12:**
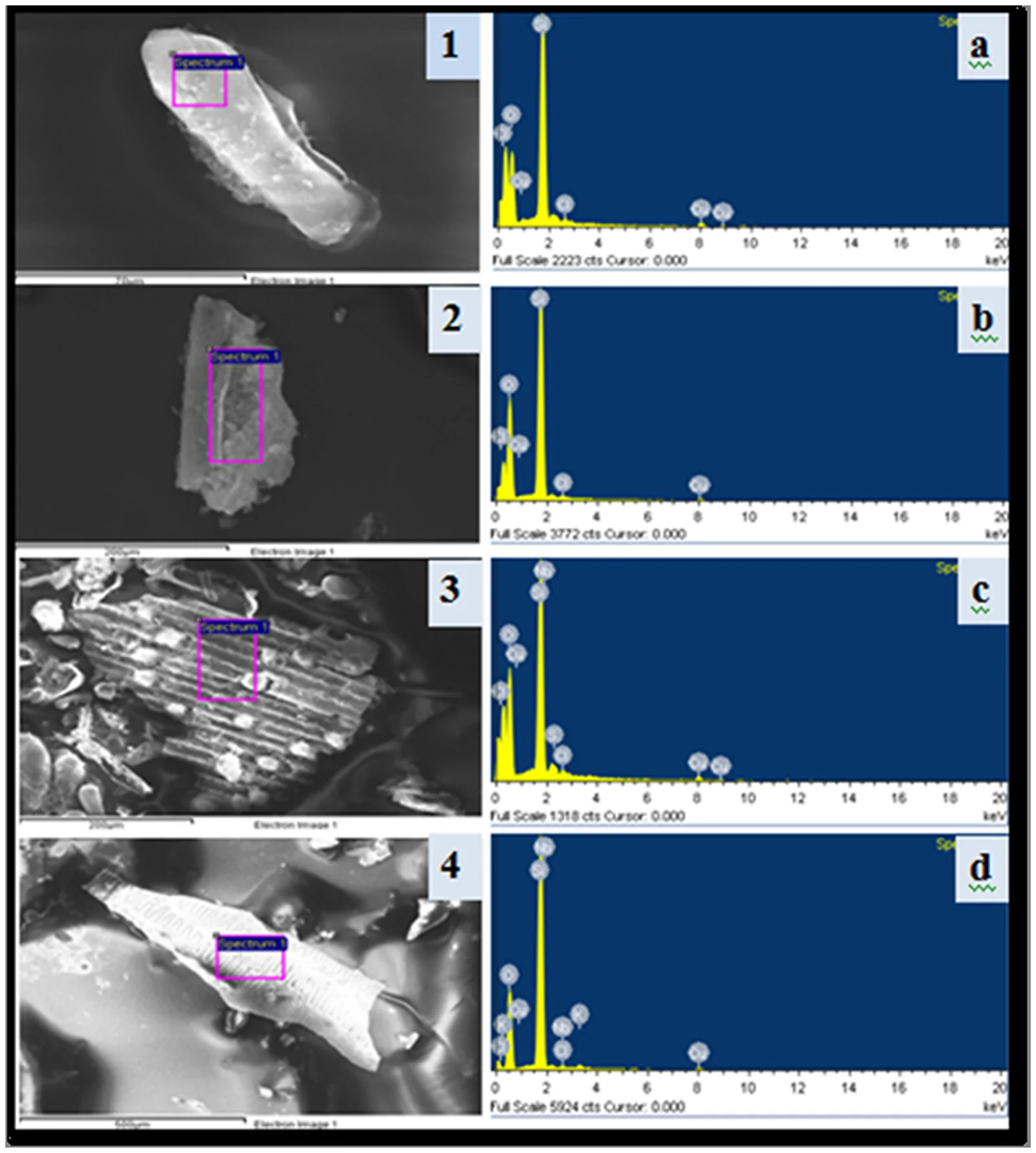
Elemental composition spectra of phytolith morphotypes of *Echinochloa colona* **(1a)** root; **(2b)** culm; **(3c)** leaf; **(4d)** inflorescence.

**Figure 13:**
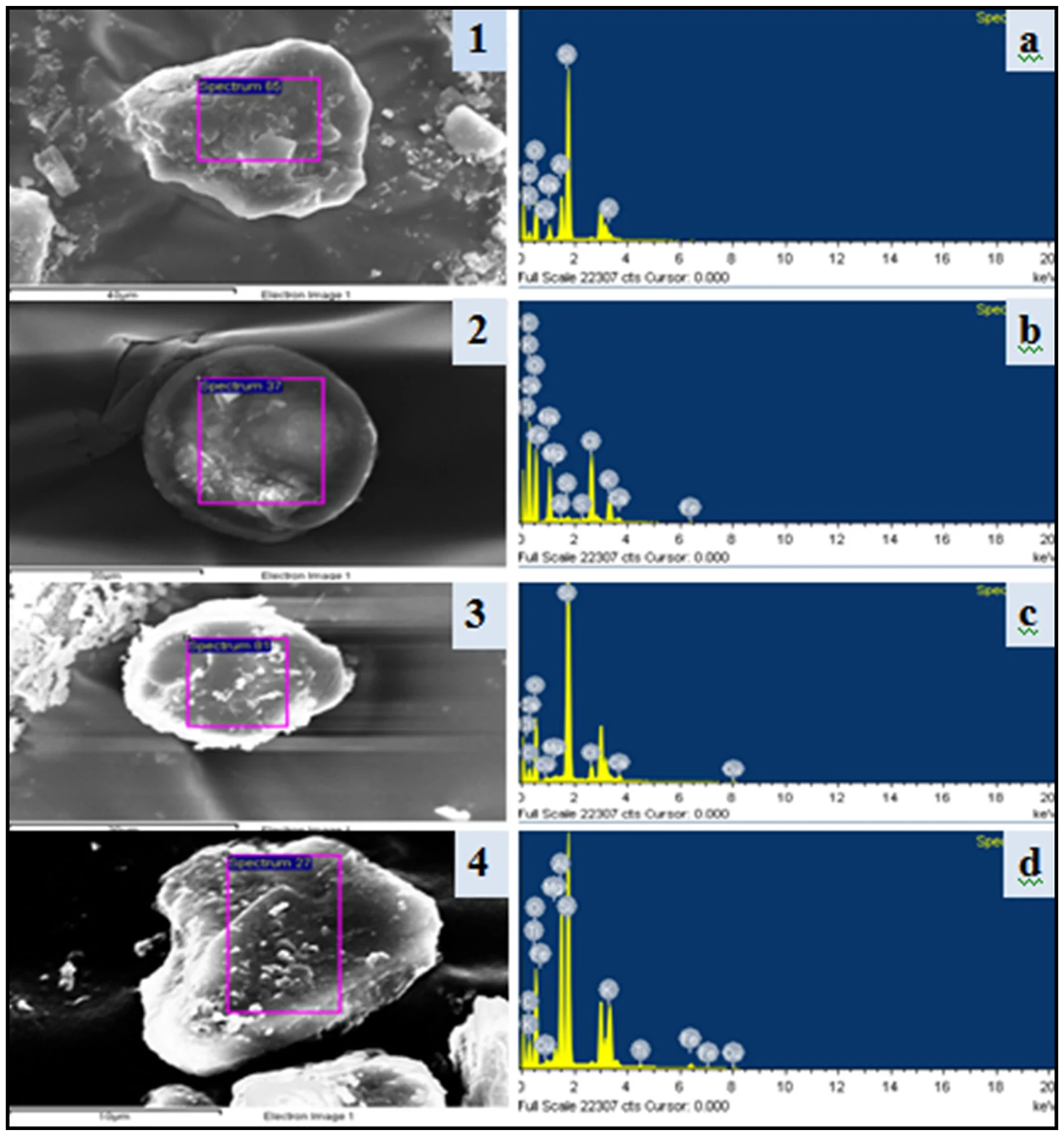
Elemental composition spectra of phytolith morphotypes of *Echinochloa crus-galli* **(1a)** root; **(2b)** culm; **(3c)** leaf; **(4d)** inflorescence.

Mn and Rb were detected in the analysed spectra of *E. colona*, whereas N, S and Ti were detected in the analysed spectra of *E. crus-galli*. These elements occurred at relatively lower concentrations than the principal elements. The complete elemental composition of the analysed phytolith preparations is presented in **Table 2**.

Principal component analysis based on the elemental composition data showed that the first two principal components accounted for 97.12% of the total variation, with PC1 explaining 85.12% and PC2 explaining 12.00% (**Figure 14**). The elemental profiles of the analysed *E. colona* and *E. crus-galli* samples were separated in the PCA ordination.

**Figure 14:**
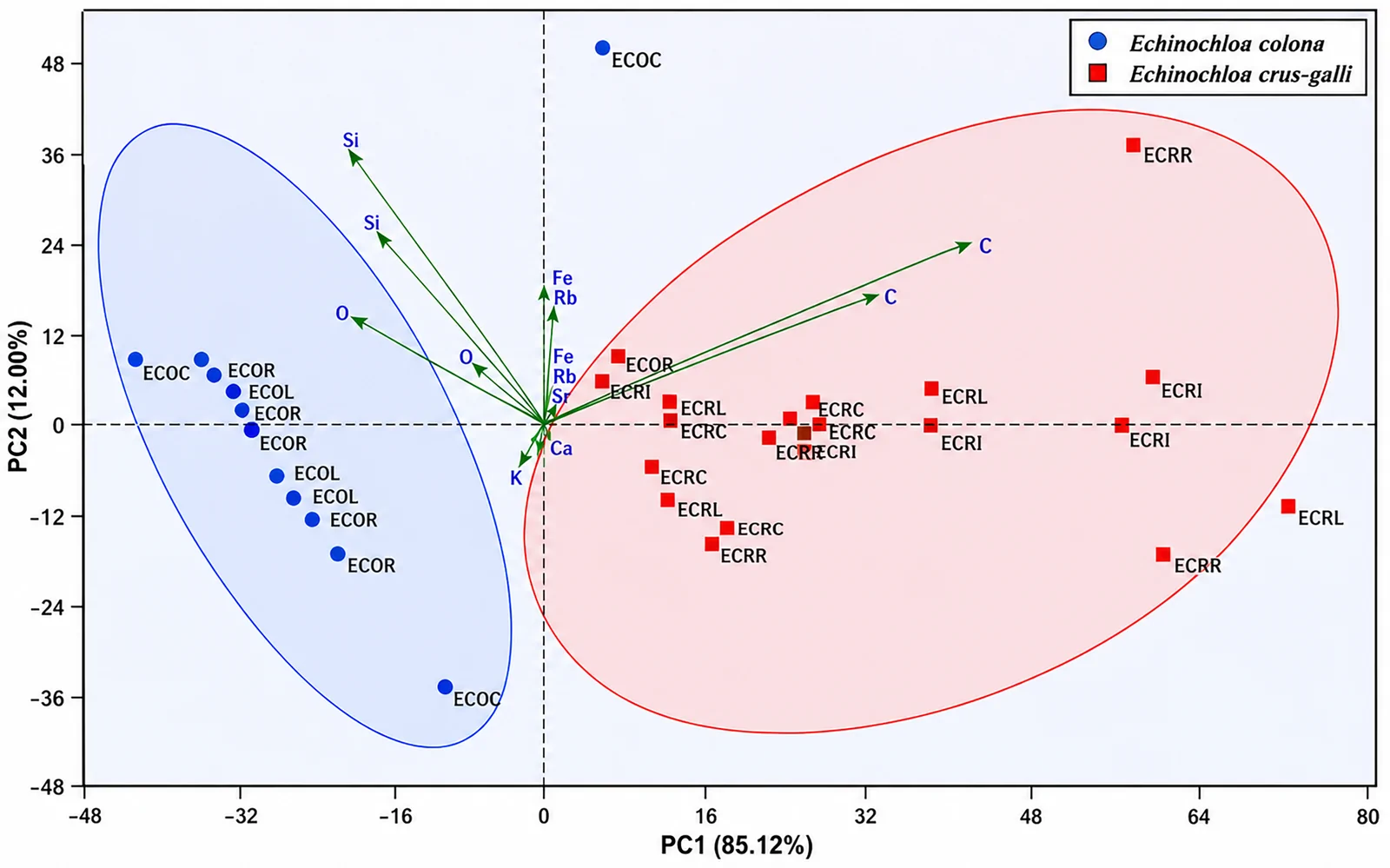
Principal component analysis (PCA) plot of phytoliths based upon their elemental composition.(ECOR, *Echinochloa colona* root; ECOC, *Echinochloa colona* culm; ECOL, *Echinochloa colona* leaf; ECOI, *Echinochloa colona* inflorescence; ECRR, *Echinochloa crus-galli* root; ECRC, *Echinochloa crus-galli* culm; ECRL, *Echinochloa crus-galli* leaf; ECRI, *Echinochloa crus-galli* inflorescence).

Carbon showed the strongest positive loading along PC1, whereas silicon and oxygen showed negative loadings. The loading values and contributions of the individual elements are presented in the corresponding PCA output.

## 4 Discussion

### 4.1 Foliar epidermal variation and silica-cell organization

The organization of leaf epidermal cells, particularly the distribution and morphology of silica cells, has long been recognized as an important source of taxonomic information in grasses and has been widely employed for species- and genus-level discrimination (Prat, 1936; Barthlott and Martens, 1979; Bhat et al., 2018; Naskar and Bera, 2018; Badgal et al., 2022). Grass leaves typically exhibit a dimorphic epidermal architecture consisting of longitudinal files of epidermal long and short cells arranged within distinct costal and intercostal zones (Metcalfe, 1960; Rudall et al., 2014; Chaudhary et al., 2023a; 2023b). Silica deposition is commonly concentrated within epidermal short cells associated with vascular bundles, giving rise to diverse grass silica short-cell phytoliths (GSSCPs), including bilobates, polylobates, crosses, nodular bilobates, saddles, rondels and trapezoids (Piperno, 2006; Neumann et al., 2019; Attolini et al., 2023; Biswas et al., 2024).

The concentration of silica phytoliths in the costal regions of both *Echinochloa colona* and *E. crus-galli* is therefore consistent with the general pattern of epidermal silicification in grasses. However, the two taxa differed in the composition and spatial organization of silica-cell morphotypes. In particular, the occurrence of saddle and convex-lobed bilobate morphotypes on the adaxial surface of *E. crus-galli*, together with the occurrence of flat-lobed bilobates in *E. colona*, indicates differences in the developmental expression and organization of silica short cells between the two species.

The differences observed on the abaxial epidermis provide an additional level of variation. The comparatively abundant and regularly arranged silica–cork cell pairs in *E. colona*, together with uneven-lobed bilobates and polylobates, contrasted with the simpler silica-cell organization observed in the analysed material of *E. crus-galli*. Such variation is potentially important because phytolith morphology reflects the dimensions and developmental characteristics of the cells in which silica is deposited. Consequently, differences in silica-cell organization may contribute to the contrasting phytolith assemblages recovered from the two species.

These observations suggest that the spatial distribution of GSSCP morphotypes may provide complementary information for distinguishing the two focal taxa. However, because the present investigation examined populations from a single locality, the observed epidermal differences should be regarded as potentially discriminating characteristics of the sampled populations rather than universally invariant species-level characters. Future comparisons involving multiple populations and contrasting habitats will be necessary to establish the geographical and environmental stability of these epidermal patterns.

### 4.2 Organ-specific ash and silica accumulation

Silicon accumulation in grasses commonly exhibits pronounced organ-specific variation, with leaves frequently functioning as major sites of silica deposition, followed by reproductive structures and other vegetative organs (Ma and Takahashi, 2002; Hodson et al., 2005; Piperno, 2006). Such differential allocation influences the quantity and composition of phytoliths produced by individual organs and therefore contributes to the development of organ-specific phytolith assemblages.

The observed differences in ash content between organs and species reflect variation in total mineral accumulation and biomass composition. The relatively high ash content observed in the culm of *E. crus-galli* may be associated with differences in total inorganic constituents and structural biomass. Importantly, however, ash content does not necessarily correspond directly with silica concentration, as demonstrated by the contrasting patterns observed among the analysed organs.

Both *Echinochloa* species exhibited maximum silica concentrations in leaves, followed by inflorescences, roots and culms. This pattern is consistent with the preferential transport of soluble silicon towards transpiring aerial tissues. Silicon absorbed by roots as silicic acid is transported through the transpiration stream and subsequently polymerizes into amorphous silica within and between specific cells and tissues. The high silica concentration in leaves may therefore reflect the combined effects of transpiration and the development of specialized silica-producing epidermal cells.

Foliar silica accumulation is considered to contribute to mechanical rigidity, leaf erectness and protection against herbivores and pathogenic organisms (Ma and Yamaji, 2015; Coskun et al., 2019). Similarly, silica deposition in inflorescences may contribute to the mechanical reinforcement and protection of reproductive structures such as glumes, lemmas and paleas (Soukup and Elbaum, 2017; Mandlik et al., 2020; Xu et al., 2023). The comparatively lower concentrations observed in roots and culms may reflect differences in the physiological functions and patterns of silicon allocation among these organs.

The higher silica concentrations observed in the leaves and inflorescences of the analysed *E. crus-galli* material suggest differences in silicon uptake, translocation or deposition relative to *E. colona*. Such differences may influence both the abundance and diversity of phytolith morphotypes produced by different organs. Nevertheless, silicon accumulation is influenced by several interacting factors, including soil silicon availability, plant physiology and environmental conditions. Therefore, the observed interspecific differences should be interpreted cautiously until they are evaluated across multiple populations and environmental conditions.

### 4.3 Diversity, organ-wise distribution and taxonomic significance of phytolith morphotypes

Phytoliths isolated from different plant organs exhibit considerable variation in morphology, size and abundance and therefore constitute a potentially useful source of taxonomic information at the family, genus and species levels (Bhat et al., 2018; Badgal et al., 2022; Chaudhary et al., 2023a; 2023b; Morcote-Rios et al., 2025). Nevertheless, many phytolith studies have focused predominantly on leaves and inflorescences, whereas roots and other vegetative organs have received comparatively less attention (Hodson and Sangster, 1989; Kumar et al., 2017a; Chen et al., 2020). Earlier investigations have demonstrated the taxonomic value of foliar phytolith assemblages in grasses, including *Echinochloa*, while studies of reproductive structures have shown that inflorescence-derived phytoliths can also provide useful discriminatory information (Ball et al., 1996; Krishnan et al., 2000; Ge et al., 2020).

The present study extends this comparative framework by examining phytolith assemblages from roots, culms, leaves and inflorescences of *E. colona* and *E. crus-galli*. A total of fifty morphotypes were recorded across the two species, demonstrating substantial variation in phytolith production among organs and between taxa. The greater number of morphotypes recorded in *E. crus-galli* than in *E. colona* suggests differences in the diversity of silicified anatomical structures represented in the analysed material. However, morphotype richness should not be regarded as an invariant species-level character, because phytolith production may also vary with developmental stage, plant physiology and environmental conditions.

The distribution of morphotypes among roots, culms, leaves and inflorescences further highlights the importance of organ-wise sampling. Phytolith diversity was not confined to the conventionally investigated leaves and reproductive structures; roots and culms also contributed distinctive morphotypes to the overall assemblage. This supports the value of sampling both below-ground and above-ground organs when characterising phytolith diversity in grasses. Comparable organ-wise investigations have reached similar conclusions. Shakoor et al. (2016), for example, documented taxonomically informative phytolith assemblages in the roots, culms, leaves and inflorescences of *Arundo donax* and *Phragmites australis*. Likewise, studies of *Acrachne racemosa*, *Lolium perenne* and *Rostraria cristata* have shown that different plant organs can contribute complementary phytolith characters (Badgal et al., 2022; Chowdhary et al., 2022; Chowdhary et al., 2023). The present findings are therefore consistent with the broader recognition that organ-level variation should be incorporated into phytolith reference collections and comparative taxonomic studies.

The ubiquity values and diagnostic index provide a quantitative basis for evaluating the distribution and potential discriminatory value of individual morphotypes within the present dataset. Morphotypes restricted to one of the two focal species, or confined to particular organs, have greater comparative value than forms that are widely distributed across both taxa and multiple organs. Conversely, morphotypes with high ubiquity values are less useful as individual diagnostic characters because of their broad occurrence. This pattern indicates that the taxonomic utility of a morphotype depends not only on its morphology but also on the consistency and specificity of its distribution.

Several morphotypes showed restricted occurrence within the analysed material and therefore represent potentially useful discriminatory characters. In *E. colona*, these included acicular, bilobate with long shank and convex lobes, cuboid, dendritic, fusiform, rectangular, rectangular with columellate extensions, rondel keeled, spheroid granulate, spheroid polyhedral and tabular irregular forms. In contrast, blocky polyhedral, blocky rectangular, cross, cylindrical, elongate dendritic, elongate smooth, tracheid, M-shaped nodular bilobate, orbicular, parallelepipedal bulliform cell, polylobate irregular, rondel (elongate notched type), rondel complex, saddle, saddle (narrow/deformed type), tabular tracheid and trapezoid keeled tracheid were restricted to the analysed *E. crus-galli* material. These species-restricted occurrences increase the discriminatory potential of the assemblages, particularly when considered together with organ-specific distribution rather than as isolated characters.

The hierarchical cluster analysis based on presence–absence data further demonstrated marked differences in phytolith assemblage composition between *E. colona* and *E. crus-galli*. Similarity between corresponding organs was highest for the leaf and inflorescence assemblages, whereas root assemblages showed very low similarity, indicating that the degree of phytolith resemblance between the two species is strongly organ dependent. The low overall Jaccard similarity (J = 0.229) further indicates limited overlap in organ-specific morphotype occurrence between the two taxa. These results support the use of assemblage-level presence–absence patterns as an additional source of taxonomic information and suggest that retaining organ identity provides a more informative basis for comparison than pooling phytoliths across the entire plant.

The potentially diagnostic morphotypes identified here should, however, be interpreted cautiously. The two species were sampled from agricultural fields at the same locality, which provides a broadly comparable environmental setting, but the study was restricted to a single locality. Consequently, the observed differences cannot be regarded as universally species-specific, nor can genetic differentiation be fully separated from possible effects of soil conditions, silicon availability, microenvironment and other sources of phenotypic plasticity. Multi-population studies across contrasting habitats will therefore be necessary to determine the stability of these morphotype distributions and to assess whether the presently identified characters retain their discriminatory value across broader geographical and environmental ranges.

### 4.4 Taxonomic significance of inflorescence epidermal ornamentation

The epidermal surfaces of inflorescence bracts, including glumes, lemmas and paleas, often undergo extensive silicification and may preserve distinctive patterns of long-cell undulation and surface ornamentation (Kealhofer et al., 2015; Ge et al., 2020; Le Moyne et al., 2023). Because reproductive structures frequently exhibit well-developed and anatomically distinct epidermal patterns, they can provide valuable complementary information for phytolith-based identification. The differences observed between *E. colona* and *E. crus-galli* in the analysed inflorescence bracts are therefore noteworthy. The occurrence of ∩- and η-type ornamentations in *E. colona*, in contrast to β-type ornamentation in the analysed *E. crus-galli* material, indicates differences in the silicified configuration of epidermal long cells. The subdivision of η- and β-type patterns into further subtypes also demonstrates that variation occurs at a finer morphological scale.

Such differences may provide useful comparative characters when combined with other phytolith features. In particular, the integration of inflorescence ornamentation with isolated phytolith morphotypes may improve the characterization of the two focal taxa. However, the present observations should not be interpreted as establishing universally exclusive ornamentation patterns for the species because additional populations and congeners were not examined.

A further consideration is the application of these characters to archaeological and sedimentary assemblages. Although silicified inflorescence tissues may preserve detailed epidermal patterns, fragmentation, abrasion and other taphonomic processes can obscure diagnostic surface characteristics. Consequently, the application of these ornamentation patterns to fossil material should rely on comparisons with well-developed modern reference collections and should preferably be integrated with multiple phytolith characters rather than based on a single diagnostic feature.

### 4.5 Quantitative significance of phytolith frequency and morphometric variation

Historically, phytolith identification has relied largely on qualitative descriptions of morphology. However, closely related taxa often produce broadly similar phytolith morphotypes, limiting the discriminatory power of morphology alone. Quantitative approaches based on morphotype frequencies and morphometric measurements can therefore provide an important complementary basis for taxonomic differentiation (Strömberg, 2009; Jattisha and Sabu, 2012; Tripathi et al., 2013; Out et al., 2014; Ball et al., 2016; Shakoor et al., 2016; Naskar and Bera, 2018). In the present study, this was evident from the significant differences in the relative frequencies of several morphotypes shared by *Echinochloa colona* and *E. crus-galli*. For instance, prickle hair and trapezoid phytoliths occurred in both species but differed significantly in abundance, indicating that taxonomic information may be conveyed not only by the presence or absence of particular morphotypes but also by their proportional representation within phytolith assemblages.

This distinction is particularly important when interpreting sedimentary or archaeological phytolith assemblages, where individual morphotypes are commonly shared among several taxa. Under such circumstances, assemblage composition and relative abundance may provide additional discriminatory information when unique diagnostic morphotypes are absent. Nevertheless, frequency-based interpretation requires standardised counting procedures and adequate biological replication, as phytolith production and representation may vary among plant organs, developmental stages and environmental conditions.

Morphometric variation provided an additional level of discrimination between the two species. Shared morphotypes differed quantitatively in size and shape parameters, demonstrating that morphologically similar phytoliths can retain measurable interspecific differences. The larger mean area of spheroid irregular phytoliths in *E. crus-galli*, together with differences in the dimensions of nodular bilobates, illustrates the potential value of quantitative measurements for distinguishing otherwise similar morphotypes. Measurements such as length, width, area, perimeter and aspect ratio characterise different components of phytolith geometry, while additional traits such as shank length and width in bilobate forms can further resolve variation within broadly similar morphological categories. Such quantitative descriptors reduce dependence on subjective visual assessment and allow phytolith differences to be evaluated statistically (Ball et al., 2016).

The discriminant function analysis further supported the taxonomic potential of phytolith morphometry. When comparisons were restricted to morphotypes shared by both species within the same plant organ, the analysis revealed significant separation between *E. colona* and *E. crus-galli*. Width contributed most strongly to the discriminant function, followed by area and perimeter, suggesting that lateral dimensions and overall phytolith size may carry greater interspecific information than length alone. The strongest separation was associated mainly with leaf and inflorescence morphotypes, including macrohair, scutiform, *Stipa*-type bilobate and other bilobate forms, indicating that these organs may contain particularly informative morphometric characters. However, the decline in classification success following cross-validation shows that this separation was not absolute. The discriminant results should therefore be interpreted as supportive evidence of interspecific differentiation rather than as a definitive diagnostic criterion.

Taken together, the frequency and morphometric results support an integrated approach in which qualitative morphology, assemblage composition and quantitative measurements are evaluated jointly. Such a framework is likely to be more informative than reliance on any single phytolith character, particularly for closely related taxa that share many morphotypes. At the same time, the present findings should be interpreted with appropriate caution because multiple phytoliths measured from the same individual plant cannot automatically be considered independent biological replicates. Future studies should therefore incorporate independently sampled individuals and populations and assess both within- and among-population variation. Broader geographical and environmental replication will be necessary to determine whether the morphometric and frequency differences observed here remain stable enough to support reliable species-level identification.

### 4.6 Anatomical control of phytolith formation

The anatomical origin of phytolith morphotypes provides an essential framework for understanding the relationship between silica deposition and phytolith morphology. Phytoliths are formed through the deposition and polymerization of silicic acid within specific cells, cell walls and intercellular spaces, and their morphology is strongly influenced by the dimensions and developmental characteristics of the anatomical structures in which silicification occurs (Piperno, 1988, 2006; Hodson et al., 2005; Hodson, 2016; Kumar et al., 2017b; Neumann et al., 2019; Chen et al., 2020).

The correlation of isolated phytolith morphotypes with corresponding silica-producing cells in the present study therefore provides important anatomical context for interpreting the phytolith assemblages of *E. colona* and *E. crus-galli*. The involvement of epidermal long cells, silica short cells, bulliform cells, trichomes, stomatal complexes and tracheary elements demonstrates that the overall phytolith assemblage represents the combined silicification of multiple tissues.

Knowledge of anatomical origin is particularly important because similar morphological forms may originate from different tissues or may occur across multiple taxa. Consequently, identifying the parent tissue of a phytolith can improve the interpretation of its taxonomic and ecological significance. This principle is also relevant to archaeological and palaeoenvironmental applications, where isolated phytoliths are frequently recovered without their original anatomical context.

The present anatomical observations therefore strengthen the modern reference value of the dataset by linking isolated phytolith forms with their corresponding tissues and organs. However, anatomical interpretations should be confirmed wherever possible using *in situ* observations and repeated sampling because developmental and environmental variation may influence the extent and location of silicification.

Although phytoliths consist predominantly of hydrated amorphous silica, they may contain or be associated with additional elements incorporated during biomineralization or retained within the analysed phytolith preparation. Their elemental composition may be influenced by plant physiology, soil geochemistry, nutrient availability and environmental conditions (Kameník et al., 2013; Bujan, 2013; Hodson, 2016; Tan et al., 2022; Pokrovsky et al., 2024).

The detection of fourteen elements in the analysed *E. colona* preparations and fifteen elements in *E. crus-galli* indicates substantial elemental diversity. The predominance of silicon and oxygen is consistent with the fundamentally siliceous nature of phytoliths and reflects the deposition of amorphous hydrated silica.

The detection of carbon is also of interest because carbon can become associated with phytoliths during plant biomineralization. Previous studies have described the presence of phytolith-occluded carbon resulting from the entrapment of organic carbon during silica deposition (Parr and Sullivan, 2005; Hodson, 2016; Pokrovsky et al., 2024). In the present study, the repeated detection of carbon is therefore consistent with the possibility of carbon associated with the silica matrix. However, SEM–EDX detects elemental carbon and does not independently establish its chemical form or precise structural location.

This limitation is particularly important because carbon analysis requires rigorous purification procedures. The use of double digestion and repeated cleaning was intended to minimize the contribution of organic and mineral impurities. Nevertheless, the possibility of residual contamination or methodological artefacts cannot be completely excluded. Carbon detected by SEM–EDX should therefore be interpreted cautiously and should not be regarded as a definitive quantitative measure of phytolith-occluded carbon without complementary analytical techniques.

The detection of Mn and Rb in the analysed spectra of *E. colona*, and N, S and Ti in *E. crus-galli*, provides additional evidence of variation in the elemental profiles of the two sampled populations. However, these elements occurred at relatively low concentrations and should not presently be regarded as universally exclusive species-level elemental markers. Trace elements can be influenced by soil conditions, plant nutrient uptake, sample preparation and analytical detection limits. Their taxonomic significance therefore requires validation through repeated analysis of multiple populations and independently collected material.

Several additional elements, including aluminium, iron, copper, potassium, calcium and magnesium, were consistently detected in the phytolith preparations. Their occurrence may reflect the interaction between silica biomineralization and mineral uptake, sequestration or compartmentalization. Silicon has been associated with plant tolerance to a range of abiotic stresses, including metal toxicity and salinity (Okuda and Takahashi, 1962; Matoh et al., 1986; Cocker et al., 1998; Yeo et al., 1999; Anala and Nambisan, 2015; Mandlik et al., 2020; Xu et al., 2023).

Nevertheless, the precise origin and physiological significance of individual minor elements cannot be determined from SEM–EDX data alone. Additional techniques capable of determining chemical state and spatial distribution would be required to establish whether these elements are structurally incorporated into the phytolith matrix, associated with surface residues or derived from other components of the preparation.

The PCA based on elemental composition data provided a multivariate visualization of the differences between the analysed elemental profiles of *E. colona* and *E. crus-galli*. The high proportion of variation explained by PC1 and PC2 indicates that the elemental dataset was strongly structured within the present analytical framework.

The alignment of silicon and oxygen is consistent with their shared association with the silica-dominated composition of phytoliths. In contrast, the opposing orientation of carbon relative to silica-associated elements indicates differences in the contribution of carbon to the analysed elemental profiles.

The separation of the two species in the PCA demonstrates that the sampled populations differed in their overall elemental composition. However, PCA is an exploratory dimensionality-reduction technique and does not, by itself, establish classification accuracy or universal taxonomic discrimination. Moreover, because the elemental profiles were obtained from plants collected at a single locality, environmental and edaphic effects cannot be completely separated from species-associated variation.

Elemental PCA should therefore be interpreted as complementary to morphological and morphometric evidence rather than as independent proof of species identity. Future investigations should include replicated populations from contrasting habitats and should evaluate the reproducibility of elemental profiles using complementary analytical techniques.

### 4.9 Taxonomic, archaeobotanical and palaeoenvironmental implications

The integrated dataset generated in the present study provides a modern comparative framework for examining phytolith variation in *E. colona* and *E. crus-galli*. The combination of organ-wise morphology, ultrastructural observations, relative-frequency patterns, quantitative morphometry, anatomical origin and elemental characterization offers a broader approach than reliance on qualitative phytolith morphology alone.

The results may be particularly useful for developing comparative reference collections for the identification of modern and archaeological grass assemblages. Morphotypes that show restricted occurrence within the present dataset, together with differences in frequency and morphometric characteristics of shared forms, may provide complementary information when used collectively.

However, direct application to archaeological and palaeoenvironmental samples requires caution. Sedimentary phytolith assemblages represent mixtures of material derived from multiple plant taxa and organs. In addition, dissolution, fragmentation, abrasion, corrosion and other taphonomic processes may modify phytolith morphology and alter assemblage composition. Consequently, characters that are distinct in modern plants may become less readily identifiable after deposition and long-term burial.

Elemental signatures may be particularly vulnerable to post-depositional alteration. While major silica-related components may remain relatively stable, low-abundance trace elements can potentially be modified through dissolution, adsorption or interaction with surrounding sediments. The elemental differences identified in modern phytolith preparations should therefore not be directly extrapolated to fossil phytoliths without experimental and archaeological validation.

The most reliable future application of the present findings will therefore involve the use of multiple complementary characters rather than dependence on a single morphotype or elemental signature. An assemblage-based approach integrating morphology, frequency, morphometry and, where preservation permits, chemical characteristics is likely to provide a more robust basis for interpretation.

### 4.10 Study limitations

Several limitations should be considered when interpreting the findings of the present study. First, the sampling was restricted to one population of each species collected from the same agricultural-field locality at Guru Nanak Dev University, Amritsar, Punjab. Although collecting both taxa from the same locality provided a broadly comparable environmental background, the present design does not capture geographical and population-level variation within either species. The observed phytolith characteristics should therefore be interpreted as potentially discriminating features of the sampled populations rather than universally invariant species-specific characters.

Second, environmental and edaphic influences cannot be completely excluded. Soil silicon availability, soil physicochemical properties, nutrient status, water availability and other environmental conditions may influence silicon uptake, deposition and phytolith development. These variables were not independently quantified or experimentally controlled in the present field-based investigation. Consequently, the relative contributions of genetic differentiation and environmental plasticity to the observed interspecific differences cannot be completely separated.

Third, the comparative framework was restricted to *E. colona* and *E. crus-galli*. Additional morphologically similar congeners, including *E. oryzicola* and *E. crus-pavonis*, were not included. Therefore, the extent to which the observed phytolith characters are unique to the two focal species or shared more broadly within the genus remains to be determined.

Fourth, although double digestion and repeated cleaning procedures were employed to minimize impurities before SEM–EDX analysis, the elemental profiles should be interpreted with appropriate caution. SEM–EDX cannot independently establish the precise chemical state or structural location of every detected element. This is particularly relevant to low-abundance elements and to the interpretation of carbon as phytolith-occluded carbon.

Finally, the modern phytolith characters identified in this study have not yet been validated under taphonomic or diagenetic conditions. Dissolution, fragmentation, abrasion and post-depositional chemical alteration may modify phytolith morphology, relative abundance and elemental composition in archaeological and sedimentary contexts. Consequently, the application of the present reference data to ancient materials requires further experimental and empirical validation.

### 4.11 Future prospects

Future investigations should expand the present comparative framework by incorporating multiple populations of each species across contrasting geographical regions, habitats and soil conditions. Measurements of soil physicochemical properties, particularly plant-available silicon, should be integrated with phytolith analyses to evaluate the contribution of environmental conditions to phytolith variation.

Controlled-environment or common-garden experiments would provide a particularly useful approach for distinguishing genetically associated variation from environmentally induced plasticity. Such studies could determine whether the morphological, morphometric and elemental differences observed between *E. colona* and *E. crus-galli* remain stable when the two species are grown under comparable conditions.

Future comparative investigations should also include additional morphologically similar *Echinochloa* congeners. Expanding the taxonomic reference framework will be essential for determining whether the potentially diagnostic characters identified in the present study are restricted to the two focal species or occur more broadly within the genus.

The quantitative framework may also be strengthened through multivariate classification approaches. Discriminant analysis can be used to evaluate the classification accuracy of selected phytolith morphometric variables, while larger independently replicated datasets could subsequently support more advanced supervised approaches. Candidate feature sets may include phytolith length, width, area, perimeter, aspect ratio, relative frequencies of selected morphotypes and reproducible elemental variables. Models should be validated using independently sampled plants and, where possible, geographically independent populations to avoid overestimation of classification accuracy.

Finally, experimental weathering and burial studies, together with analyses of archaeological and sedimentary phytolith assemblages, are required to determine which modern diagnostic characters persist after taphonomic and diagenetic alteration. Such investigations will be essential for translating modern phytolith reference data into reliable applications in archaeobotany and palaeoenvironmental reconstruction.

Overall, the present study provides an integrated organ-wise comparative framework for investigating phytolith variation in *E. colona* and *E. crus-galli*. Its principal contribution lies in the combined evaluation of phytolith morphology, quantitative morphometry, frequency distributions, anatomical origin and elemental characteristics. Future multi-population, multi-species and experimentally controlled studies will be required to determine the broader taxonomic stability and environmental robustness of these characters and to establish their applicability to archaeological and palaeoenvironmental phytolith assemblages.

## 5 Conclusion

The present study demonstrates that phytolith-based discrimination within *Echinochloa* is most effective when phytolith characters are interpreted as an integrated assemblage rather than as isolated diagnostic features. The combined evidence from organ-specific morphology, epidermal silicification, morphotype distribution, relative abundance, morphometry, ultrastructure and elemental composition indicates that *E. colona* and *E. crus-galli* differ across several complementary dimensions of phytolith expression. Importantly, these differences also reveal that the taxonomic information carried by phytoliths is strongly dependent on plant organ, reinforcing the need for reference collections that retain anatomical and organ-specific context rather than pooling material from the whole plant.

The multivariate analyses further support this integrated perspective. Presence–absence relationships demonstrate that assemblage composition itself contains useful discriminatory information, whereas morphometric analysis shows that quantitative variation can refine distinctions among morphotypes that are otherwise shared between the two species. However, the moderate cross-validated discrimination also indicates that morphometry should be regarded as supportive rather than independently diagnostic. Accordingly, the strongest taxonomic inference emerges from convergence among qualitative, quantitative and compositional phytolith characters.

Beyond species identification, the study provides a useful modern comparative framework for interpreting *Echinochloa* phytoliths in archaeobotanical, palaeoecological and sedimentary assemblages. Such applications should nevertheless avoid reliance on single morphotypes or elemental signatures, particularly because preservation, environmental conditions and developmental plasticity may modify their expression.

The principal limitation of the present investigation is its restriction to one population of each species from a single locality. The characters identified here should therefore be regarded as potentially discriminating rather than universally species-specific. Future work incorporating multiple populations, additional congeners, controlled environmental experiments and independent biological replication will be essential for distinguishing genetically stable taxonomic signals from environmentally induced variation and for establishing the broader reliability of phytolith-based species identification within *Echinochloa*.

## Data Availability

The raw data supporting the conclusions of this article will be made available by the authors, without undue reservation.
