# Supplementary material for: Comparative phytolith profiles of the weed grasses Echinochloa colona and Echinochloa crus-galli (Poaceae): implications for species taxonomy
Source: Front Plant Sci. 2026 Oct 28;17:1964606. doi: 10.3389/fpls.2026.1964606 (PMC13751965; doi:10.3389/fpls.2026.1964606)
Supplement: Supplementary file 1 [file Table1.doc]

**Supplementary Table 1. Description of different phytolith morphotypes and their diagnostic potential in *Echinochloa* spp. of the present study.**

| **S.No** | **Phytolith Morphotypes** | **Acronym** | **Morphotype description (as per ICPN1.0)** | | | ***Echinochloa colona*** | | | | ***Echinochloa crus-galli*** | | | | **Diagnosticfor** | **Ubiquity**  **Species (Organ)** |
| --- | --- | --- | --- | --- | --- | --- | --- | --- | --- | --- | --- | --- | --- | --- | --- |
|  |  |  | **First descriptor** | **Second descriptor** | **Third descriptor** |  |  |  |  |  |  |  |  |  |  |
|  |  |  |  |  |  | **Rt** | **Cl** | **Lf** | **Inflo** | **Rt** | **Cl** | **Lf** | **Inflo** |  |  |
| 1. | Acicular | ACI | Acicular | ---------------- | ---------------- | ----------- | ----------- | + | ----------- | ----------- | ----------- | ----------- | ----------- | **ECO** | **0.50(12.5)** |
| 2. | Bilobate with long shank and concave lobes | LOB_CLA-I | Lobate class I | ---------------- | ---------------- | + | ----------- | + | ----------- | ----------- | ----------- | ----------- | + | **------** | **1.00(37.5)** |
| 3. | Bilobate with long shank and convex lobes | LOB_CLA-I | Lobate class II | ---------------- | ---------------- | ----------- | ----------- | + | ----------- | ----------- | ----------- | ----------- | ----------- | **ECO** | **0.50(12.5)** |
| 4. | Bilobate with short shank and concavel obes | LOB_CLA-I | Lobate class III | ---------------- | ---------------- | ----------- | ----------- | ----------- | + | + | ----------- | ----------- | ----------- | **------** | **1.00(25.0)** |
| 5. | Bilobate with short shank and convex obes | LOB_CLA-I | Lobate class IV | ---------------- | ---------------- | ----------- | ----------- | + | ----------- | ----------- | ----------- | + | ----------- | **------** | **1.00(25.0)** |
| 6. | Bilobate with short shank and flat lobes | LOB_CLA-I | Lobate class V | ---------------- | ---------------- | ----------- | ----------- | + | ----------- | + | ----------- | + | ----------- | **------** | **1.00(37.5)** |
| 7. | Bilobate with uneven lobes | LOB_CLA-I | Lobate class VI | ---------------- | ---------------- | ----------- | ----------- | ----------- | + | ----------- | ----------- | ----------- | + | **------** | **1.00(25.0)** |
| 8. | Blocky irregular | BLO_IRR | Blocky | Irregular | ---------------- | + | ----------- | ----------- | + | + | ----------- | ----------- | + | **------** | **1.00(50.0)** |
| 9. | Blocky polyhedral | BLO_POL | Blocky | polyhedral | ---------------- | ----------- | ----------- | ----------- | ----------- | + | ----------- | ----------- | + | **ECR** | **0.50(25.0)** |
| 10. | Blocky rectangular | BLO_REC | Blocky  rectangular | ---------------- | ---------------- | ----------- | ----------- | ----------- | ----------- | ----------- | ----------- | + | ----------- | **ECR** | **0.50(12.5)** |
| 11. | Clavate | CLA | Clavate | ---------------- | ---------------- | ----------- | ----------- | ----------- | + | + | ----------- | ----------- | ----------- | **------** | **1.00(25.0)** |
| 12. | Cross | CRO | Cross | ---------------- | ---------------- | ----------- | ----------- | ----------- | ----------- | ----------- | + | ----------- | ----------- | **ECR** | **0.50(12.5)** |
| 13. | Cuboid | CUB | Cuboid | ---------------- | ---------------- | ----------- | + | ----------- | + | ----------- | ----------- | ----------- | ----------- | **ECO** | **0.50(25.0)** |
| 14. | Cuneiform bulliformcell | CUN_BUL | Cuneiform | ---------------- | Bulliform cell | ----------- | ----------- | + | ----------- | ----------- | ----------- | + | ----------- | **------** | **1.00(25.0)** |
| 15. | Cylindrical | CYL | Cylindrical | ---------------- | ---------------- | ----------- | ----------- | ----------- | ----------- | ----------- | ----------- | + | ----------- | **ECR** | **0.50(12.5)** |
| 16. | Dendritic | DEN | Dendritic | ---------------- | ---------------- | ----------- | ----------- | ----------- | + | ----------- | ----------- | ----------- | ----------- | **ECO** | **0.50(12.5)** |
| 17. | Elongate dendritic | ELO_DEN | Elongate | Dendritic | ---------------- | ----------- | ----------- | ----------- | ----------- | ----------- | + | + | ----------- | **ECR** | **0.50(25.0)** |
| 18. | Elongate ridged | ELO_RID | Elongate | Ridged | ---------------- | ----------- | + | ----------- | ----------- | + | ----------- | ----------- | ----------- | **------** | **1.00(50.0)** |
| 19. | Elongate smooth | ELO_SMO | Elongate | Smooth | ---------------- | ----------- | ----------- | ----------- | ----------- | ----------- | + | + | ----------- | **ECR** | **0.50(25.0)** |
| 20. | Elongate trachied | TRA | ---------------- | ---------------- | Trachied | ----------- | ----------- | ----------- | ----------- | + | ----------- | ----------- | ----------- | **ECR** | **0.50(12.5)** |
| 21. | Fusiform | FUS | Fusiform | ---------------- | ---------------- | ----------- | ----------- | ----------- | + | ----------- | ----------- | ----------- | ----------- | **ECO** | **0.50(12.5)** |
| 22. | Macrohairs | MAC | ---------------- | ---------------- | Macrohairs | ----------- | ----------- | ----------- | + | ----------- | ----------- | ----------- | + | **------** | **1.00(25.0)** |
| 23. | M-shaped nodular bilobate | LOB_CLA-I | Lobate class | ---------------- | ---------------- | ----------- | ----------- | ----------- | ----------- | + | ----------- | + | ----------- | **ECR** | **0.50(25.0)** |
| 24. | Nodular bilobate | LOB_CLA-I | Lobate class | ---------------- | ---------------- | ----------- | ----------- | + | ----------- | ----------- | + | + | + | **------** | **1.00(50.0)** |
| 25. | Orbicular | ORB | Orbicular | ---------------- | ---------------- | ----------- | ----------- | ----------- | ----------- | + | ----------- | ----------- | ----------- | **ECR** | **0.50(12.5)** |
| 26. | Oval | OVL | Oval | ---------------- | ---------------- | + | ----------- | + | + | ----------- | ----------- | ----------- | ----------- | **ECO** | **0.50(37.5)** |
| 27. | Parallepipedal | PAR | Parallepipedal | ---------------- | ---------------- | ----------- | + | + | ----------- | + | + | + | + | **------** | **1.00(75.0)** |
| 28. | Parallepipedal bulliform cell | PAR_BUL_CEL | Parallepipedal | ---------------- | Bulliform cell | ----------- | ----------- | ----------- | ----------- | ----------- | ----------- | + | ----------- | **ECR** | **0.50(12.5)** |
| 29. | Polylobate | POL | Polylobate | ---------------- | ---------------- | ----------- | ----------- | ----------- | ----------- | ----------- | ----------- | + | ----------- | **ECR** | **0.50(12.5)** |
| 30. | Polylobate irregular | POL_IRR | Polylobate | irregular | ---------------- | ----------- | ----------- | ----------- | ----------- | ----------- | ----------- | + | ----------- | **ECR** | **0.50(12.5)** |
| 31. | Prickle hair | PRI_HAI | ---------------- | ---------------- | Prickle hair | ----------- | ----------- | ----------- | + | ----------- | ----------- | ----------- | + | **------** | **1.00(25.0)** |
| 32. | Rectangular | REC | Rectangular | ---------------- | ---------------- | ----------- | ----------- | ----------- | + | ----------- | ----------- | ----------- | ----------- | **ECO** | **0.50(12.5)** |

| 33. | Rectangular with columellate extensions | REC_COL_EXT | Rectangular | columellate  extensions | ---------------- | ----------- | ----------- | ----------- | + | ----------- | ----------- | ----------- | ----------- | **ECO** | **0.50(12.5)** |
| --- | --- | --- | --- | --- | --- | --- | --- | --- | --- | --- | --- | --- | --- | --- | --- |
| 34. | Rondel (elongate notched type) | RON_ELO_NOT | Rondel |  |  | ----------- | ----------- | ----------- | ----------- | + | ----------- | ----------- | ----------- | **ECR** | **0.50(12.5)** |
| 35. | Rondel Complex | RON_COM | Rondel | Complex | ---------------- | ----------- | ----------- | ----------- | ----------- | ----------- | ----------- | ----------- | + | **ECR** | **0.50(12.5)** |
| 36. | Rondel Keeled | RON_KEE | Rondel | Keeled | ---------------- | ----------- | + | ----------- | ----------- | ----------- | ----------- | ----------- | ----------- | **ECO** | **0.50(12.5)** |
| 37. | Saddle | SAD | Saddle | ---------------- | ---------------- | ----------- | ----------- | ----------- | ----------- | ----------- | ----------- | ----------- | + | **ECR** | **0.50(12.5)** |
| 38. | Saddle (narrow/deformed type) | SAD | Saddle | narrow/deformed |  | ----------- | ----------- | ----------- | ----------- | ----------- | + | ----------- | ----------- | **ECR** | **0.50(12.5)** |
| 39. | Scutiform | SCU | Scutiform | ---------------- | ---------------- | + | + | ----------- | + | ----------- | + | + | + | **------** | **1.00(75.0)** |
| 40. | Spheroid granulate | SPH_GRA | Spheroid | Granulate | ---------------- | + | ----------- | + | ----------- | ----------- | ----------- | ----------- | ----------- | **ECO** | **0.50(25.0)** |
| 41. | Spheroid irregular | SPH_IRR | Spheroid | Irregular | ---------------- | ----------- | ----------- | + | + | ----------- | ----------- | ----------- | + | **------** | **1.00(37.5)** |
| 42. | Spheroid polyhedral | SPH_POL | Spheroid | Polyhedral | ---------------- | ----------- | + | + | ----------- | ----------- | ----------- | ----------- | ----------- | **ECO** | **0.50(25.0)** |
| 43. | *Stipa* -type bilobate | LOB_CLA-I | Lobate class | ---------------- | ---------------- | ----------- | ----------- | + | ----------- | + | ----------- | + | ----------- | **------** | **1.00(37.5)** |
| 44. | Stomata | STO | ---------------- | ---------------- | Stomata | ----------- | ----------- | ----------- | ----------- | ----------- | + | ----------- | ----------- | **ECR** | **0.50(12.5)** |
| 45. | Tabular irregular | TAB_IRR | Tabular | Irregular | ---------------- | ----------- | ----------- | ----------- | + | ----------- | ----------- | ----------- | ----------- | **ECO** | **0.50(12.5)** |
| 46. | Tabular polyhedral | TAB_POL | Tabular | polyhedral | ---------------- | + | + | ----------- | + | ----------- | + | ----------- | + | **------** | **1.00(62.5)** |
| 47. | Tabular tracheid | TAB_TRA | Tabular | ---------------- | Tracheid | ----------- | ----------- | ----------- | ----------- | ----------- | + | ----------- | ----------- | **ECR** | **0.50(12.5)** |
| 48. | Towe rflat | TOW_FLAT | Tower | Flat | ---------------- | ----------- | ----------- | + | ----------- | + | ----------- | ----------- | + | **------** | **1.00(37.5)** |
| 49. | Trapezoid | TRAP | Trapezoid | ---------------- | ---------------- | + | + | + | + | ----------- | + | + | ----------- | **------** | **1.00(75.0)** |
| 50. | Trapezoid keeled tracheid | TRA_KEE_TRA | Trapezoid | Keeled | Tracheid | ----------- | ----------- | ----------- | ----------- | ----------- | + | ----------- | ----------- | **ECR** | **0.50(12.5)** |
| **TOTAL** | | | | | | **07** | **08** | **15** | **17** | **13** | **12** | **16** | **14** | **-------------** | **--------------** |

**Rt=Root; Cl=Culm ;Lf=Leaf; Inflo=Inflorescence; ECO=*Echinochloa colona*; ECR=*E. crus-galli*; (+)=Presence & (-----)= Absence of particular phytolith morphotype**

**Supplementary Table 2. Morphometric measurements of phytolith morphotypes of *Echinochloa colona.***

| **S.**  **No** | **Phytolith**  **Morphotypes** | ***Echinochloa colona*** | | | | | | | | | | | | | | | | | | | |
| --- | --- | --- | --- | --- | --- | --- | --- | --- | --- | --- | --- | --- | --- | --- | --- | --- | --- | --- | --- | --- | --- |
|  |  | **ROOT** | | | | | **CULM** | | | | | **LEAF** | | | | | **INFLORESCENCE** | | | | |
|  |  | **Length(µm)** | **Width(µm)** | **Area (µm^2^)** | **Prem(µm)** | **A/R** | **Length(µm)** | **Width(µm)** | **Area(µm^2^)** | **Prem(µm)** | **A/R** | **Length(µm)** | **Width(µm)** | **Area (µm^2^)** | **Prem(µm)** | **A/R** | **Length**  **(µm)** | **Width(µm)** | **Area(µm^2^)** | **Prem(µm)** | **Length**  **(µm)** |
| 1. | Acicular | ----------- | ----------- | ----------- | ----------- | ----------- | ----------- | ----------- | ----------- | ----------- | ----------- | 100.16±13.37 | 10.77±1.17 | 1147±305 | 224.3±28.75 | 8.94±0.60 | ----------- | ----------- | ----------- | ----------- | ----------- |
| 2. | Bilobate with long shank  And concave lobes | 31.10±0.37 | 9.40±0.75 | 301.80±7.80 | 84.73±0.66 | 2.95±0.12 | ----------- | ----------- | ----------- | ----------- | ----------- | 23.30±1.79 | 9.11±0.41 | 192.54±8.58 | 67.92±2.06 | 2.77±0.28 | ----------- | ----------- | ----------- | ----------- | ----------- |
| 3. | Bilobate with long shank  And convex lobes | ----------- | ----------- | ----------- | ----------- | ----------- | ----------- | ----------- | ----------- | ----------- | ----------- | 25.14±0.81 | 8.43±0.32 | 171.40±14.02 | 63.35±2.01 | 3.22±0.19 | ----------- | ----------- | ----------- | ----------- | ----------- |
| 4. | Bilobate with short shank  And concave lobes | ----------- | ----------- | ----------- | ----------- | ----------- | ----------- | ----------- | ----------- | ----------- | ----------- | ----------- | ----------- | ----------- | ----------- | ----------- | 15.56±1.39 | 10.41±1.12 | 169.15±10.  42 | 58.88±1.46 | 1.55±0.15 |
| 5. | Bilobate with short shank  And convex lobes | ----------- | ----------- | ----------- | ----------- | ----------- | ----------- | ----------- | ----------- | ----------- | ----------- | 17.65±1.68 | 9.45±1.01 | 169±24.05 | 57.25±3.90 | 1.92±0.19 | ----------- | ----------- | ----------- | ----------- | ----------- |
| 6. | Bilobate with short shank  And flat lobes | ----------- | ----------- | ----------- | ----------- | ----------- | ----------- | ----------- | ----------- | ----------- | ----------- | 19.70±1.05 | 9.72±0.52 | 173.41±4.12 | 61.74±1.75 | 2.27±0.12 | ----------- | ----------- | ----------- | ----------- | ----------- |
| 7. | Bilobate with uneven lobes | ----------- | ----------- | ----------- | ----------- | ----------- | ----------- | ----------- | ----------- | ----------- | ----------- | ----------- | ----------- | ----------- | ----------- | ----------- | 13.62±0.55 | 12.54±0.60 | 125.32±5.1  9 | 52.28±2.14 | 1.46±0.12 |
| 8. | Blocky irregular | 52.86±2.49 | 34.83±4.15 | 1967.80±219 | 173.27±9.82 | 1.39±0.14 | ----------- | ----------- | ----------- | ----------- | ----------- | ----------- | ----------- | ----------- | ----------- | ----------- | 64.21±8.50 | 34.52±3.18 | 2077±381 | 191.7±17.56 | 1.92±0.25 |
| 9. | Clavate | ----------- | ----------- | ----------- | ----------- | ----------- | ----------- | ----------- | ----------- | ----------- | ----------- | ----------- | ----------- | ----------- | ----------- | ----------- | 62.45±4.05 | 17.40±0.68 | 959±98.6 | 161.02±12.77 | 3.01±0.31 |
| 10. | Cuboid | ----------- | ----------- | ----------- | ----------- | ----------- | 30.46±2.09 | 18.43±1.91 | 639.7±78 | 100.44±6.62 | 1.43±0.11 | ----------- | ----------- | ----------- | ----------- | ----------- | 19.19±1.90 | 17.55±1.26 | 299.91±14.  33 | 69.76±2.09 | 1.34±0.04 |
| 11. | Cuneiform bulliform cell | ----------- | ----------- | ----------- | ----------- | ----------- | ----------- | ----------- | ----------- | ----------- | ----------- | 58.72±8.16 | 34.62±5.21 | 1882.47±485 | 175.50±26.58 | 1.50±0.11 | ----------- | ----------- | ----------- | ----------- | ----------- |
| 12. | Dendritic | ----------- | ----------- | ----------- | ----------- | ----------- | ----------- | ----------- | ----------- | ----------- | ----------- | ----------- | ----------- | ----------- | ----------- | ----------- | 43.45±6.56 | 8.57±0.69 | 432.55±94.  39 | 126.5±19.19 | 3.93±0.42 |
| 13. | Elongate ridged | ----------- | ----------- | ----------- | ----------- | ----------- | 89.92±4.58 | 16.94±1.36 | 1444.4±102 | 206.62±8.66 | 4.81±0.05 | ----------- | ----------- | ----------- | ----------- | ----------- | ----------- | ----------- | ----------- | ----------- | ----------- |
| 14. | Fusiform | ----------- | ----------- | ----------- | ----------- | ----------- | ----------- | ----------- | ----------- | ----------- | ----------- | ----------- | ----------- | ----------- | ----------- | ----------- | 39.11±2.55 | 18.94±1.44 | 541.36±55.  20 | 96.01±5.18 | 2.00±0.10 |
| 15. | Macrohairs | ----------- | ----------- | ----------- | ----------- | ----------- | ----------- | ----------- | ----------- | ----------- | ----------- | ----------- | ----------- | ----------- | ----------- | ----------- | 72.38±9.91 | 10.76±1.75 | 661.51±231 | 160.6±24.73 | 6.75±0.74 |
| 16. | Nodular bilobate | ----------- | ----------- |  | ----------- |  | ----------- | ----------- | ----------- | ----------- | ----------- | 25.27±1.75 | 6.96±0.53 | 148.47±13.75 | 65.15±2.79 | 4.2±0.77 | ----------- | ----------- | ----------- | ----------- | ----------- |
| 17. | Oval | 36.33±2.06 | 15.45±1.86 | 311.40±77.8 | 230.93±146.36 | 2.42±11.9 | ----------- | ----------- | ----------- | ----------- | ----------- | 40.92±5.40 | 20.14±2.32 | 789.60±150.3  3 | 109.95±11.05 | 1.83±0.11 | 37.78±3.11 | 19.32±2.05 | 674.29±149 | 101.87±9.60 | 1.87±0.14 |
| 18. | Parallepipedal | ----------- | ----------- | ----------- | ----------- | ----------- | 33.25±1.43 | 18.08±2.05 | 668.37±60.4 | 104.68±3.77 | 1.73±0.14 | 34.22±3.77 | 19.94±2.50 | 719.53±124.8  8 | 108.52±9.48 | 1.72±0.16 | ----------- | ----------- | ----------- | ----------- | ----------- |
| 19. | Pricklehair | ----------- | ----------- | ----------- | ----------- | ----------- | ----------- | ----------- | ----------- | ----------- | ----------- | ----------- | ----------- | ----------- | ----------- | ----------- | 39.77±5.85 | 6.51±0.91 | 263.65±81.  91 | 96.25±15.07 | 4.93±0.77 |
| 20. | Rectangular | ----------- | ----------- | ----------- | ----------- | ----------- | ----------- | ----------- | ----------- | ----------- | ----------- | ----------- | ----------- | ----------- | ----------- | ----------- | 40.98±5.28 | 13.31±1.44 | 481.75±74.  14 | 107.52±12.54 | 3.50±0.67 |
| 21. | Rectangular with  Columellate extensions | ----------- | ----------- | ----------- | ----------- | ----------- | ----------- | ----------- | ----------- | ----------- | ----------- | ----------- | ----------- | ----------- | ----------- | ----------- | 109.43±21.  62 | 42.36±3.70 | 4889±1039 | 302±43.21 | 2.43±0.41 |
| 22. | Rondel Keeled | ----------- | ----------- | ----------- | ----------- | ----------- | 26.15±2.07 | 14.04±1.12 | 390.41±24.48 | 80.71±1.91 | 1.75±0.04 | ----------- | ----------- | ----------- | ----------- | ----------- | ----------- | ----------- | ----------- | ----------- | ----------- |
| 23. | Scutiform | 47.97±5.10 | 17.47±0.72 | 724.89±109.9 | 119.30±11.50 | 2.22±0.24 | 36.08±1.81 | 16.28±1.96 | 583.08±96.47 | 103.66±8.59 | 1.98±0.28 | ----------- | ----------- | ----------- | ----------- | ----------- | 37.08±3.08 | 19.25±2.81 | 555.9±110 | 99.60±12.03 | 2.04±.125 |
| 24. | Spheroid granulate | 35.47±5.06 | 33.87±4.44 | 1147.50±316.  48 | 121.42±17.07 | 1.13±0.03 | ----------- | ----------- | ----------- | ----------- | ----------- | 26±3.55 | 21.94±3.67 | 603.08±216.5 | 88.23±14.76 | 1.18±0.04 | ----------- | ----------- | ----------- | ----------- | ----------- |
| 25. | Spheroid irregular | ----------- | ----------- | ----------- | ----------- | ----------- | ----------- | ----------- | ----------- | ----------- | ----------- | 22.50±2.75 | 20.65±2.607 | 580.708±195.  5 | 72.75±11.40 | 1.05±0.05 | 27.30±2.06 | 25.32±1.80 | 647.63±96.  18 | 96.38±8.50 | 1.12±0.01 |
| 26. | Spheroid polyhedral | ----------- | ----------- | ----------- | ----------- | ----------- | 33.57±3.17 | 28.42±2.81 | 1018.43±187.  8 | 117.59±10.79 | 1.16±0.04 | 27.34±3.75 | 27.35±3.80 | 764.30±176.3  3 | 102.26±12.14 | 1.28±0.06 | ----------- | ----------- | ----------- | ----------- | ----------- |
| 27. | *Stipa*-type bilobate | ----------- | ----------- | ----------- | ----------- | *-----------* |  |  |  |  |  | 18±1.68 | 6.96±0.47 | 127.89±8.15 | 52.71±2.51 | 2.41±0.23 | ----------- | ----------- | ----------- | ----------- | ----------- |
| 28. | Tabular irregular | ----------- | ----------- | ----------- |  |  | 53.24±10.64 | 39.59±8.45 | 2596.35±123  7 | 187.27±40 | 1.32±0.07 | ----------- | ----------- | ----------- | ----------- | ----------- | 54.88±3.49 | 41.09±3.89 | 2073±242 | 183.73±9.77 | 1.39±0.13 |
| 29. | Tabular polyhedral | 94.70±20.28 | 66.65±15.27 | 6620.12±276  8 | 296.10±62.27 | 1.45±0.12 | ----------- | ----------- | ----------- | ----------- | ----------- | ----------- | ----------- | ----------- | ----------- | ----------- | 50.25±6.22 | 24.28±1.79 | 1293±214 | 144.18±14.41 | 1.83±0.27 |
| 30. | Tower flat | ----------- | ----------- | ----------- | ----------- | ----------- | ----------- | ----------- | ----------- | ----------- | ----------- | 17.50±1.61 | 13.35±1.06 | 231.56±43.57 | 61.96±5.37 | 1.25±0.02 | ----------- | ----------- | ----------- | ----------- | ----------- |
| 31. | Trapezoid | 40.57±2.68 | 22.60±1.69 | 807.69±118 | 122.13±9.46 | 1.71±0.14 | 51.00±4.5 | 27.18±1.82 | 1170±156 | 140.71±10.80 | 1.90±0.23 | 53.38±6.70 | 32.21±4.36 | 1415±296 | 181.23±28.05 | 1.84±0.17 | 46.07±6.57 | 33.43±4.76 | 1527.15±37  3 | 151.47±19.57 | 1.41±0.09 |

The entries in the Table indicate Mean±SE &[-------]= Absence of phytolith morphotype; A/R= Aspect ratio & Prem = Perimeter

**Supplementary Table 3: Morphometric measurements of phytolith morphotypes of *Echinochloa crus-galli*.**

| **S.**  **No** | **Phytolith**  **Morphotypes** | ***Echinochloa crus-galli*** | | | | | | | | | | | | | | | | | | | |
| --- | --- | --- | --- | --- | --- | --- | --- | --- | --- | --- | --- | --- | --- | --- | --- | --- | --- | --- | --- | --- | --- |
|  |  | **ROOT** | | | | | **CULM** | | | | | **LEAF** | | | | | **INFLORESCENCE** | | | | |
|  |  | **Length(µm)** | **Width(µm)** | **Area**  **(µm^2^)** | **Prem(µm)** | **A/R** | **Length(µm)** | **Width(µm)** | **Area(µm^2^)** | **Prem(µm)** | **A/R** | **Length(µm)** | **Width(µm)** | **Area(µm^2^)** | **Prem(µm)** | **A/R** | **Length(µm)** | **Width(µm)** | **Area(µm^2^)** | **Prem(µm)** | **A/R** |
| 1. | Bilobate with long  Shank and concave lobes | ------------- | ------------- | ------------- | ------------- | ------------- | ------------- | ------------- | ------------- | ------------- | ------------- | ------------- | ------------- | ------------- | ------------- | ------------- | 20.85±1.28 | 8.43±0.72 | 158.20±11.66 | 66.79±3.69 | 2.34±0.16 |
| 2. | Bilobate with short  Shank and concave lobes | 20.29±1.00 | 11.09±0.77 | 227.92±21.74 | 68.6±4.17 | 1.89±0.15 | ------------- | ------------- | ------------- | ------------- | ------------- | ------------- | ------------- | ------------- | ------------- | ------------- | ------------- | ------------- | ------------- | ------------- | ------------- |
| 3. | Bilobate with short shank and convex lobes | ------------- | ------------- | ------------- | ------------- | ------------- | ------------- | ------------- | ------------- | ------------- | ------------- | 20.33±0.46 | 10.66±0.87 | 199.95±16.98 | 66.33±2.53 | 2.27±0.18 | ------------- | ------------- | ------------- | ------------- | ------------- |
| 4. | Bilobate with short shank and flat lobes | 20.26±2.78 | 13.49±0.91 | 253.54±32.47 | 71.19±3.81 | 1.58±0.16 | ------------- | ------------- | ------------- | ------------- | ------------- | 17.14±1.66 | 12.18±0.72 | 199.95±23.93 | 64.51±4.72 | 3.28±0.25 | ------------- | ------------- | ------------- | ------------- | ------------- |
| 5. | Bilobate with uneven lobes | ------------- | ------------- | ------------- | ------------- | ------------- | ------------- | ------------- | ------------- | ------------- | ------------- | ------------- | ------------- | ------------- | ------------- | ------------- | 16.47±0.39 | 13.57±0.59 | 215.95±12.69 | 68.13±2.31 | 1.39±0.11 |
| 6. | Blocky irregular | 58.17±4.68 | 44.97±3.17 | 2532.35±338 | 195.84±16.52 | 1.28±0.08 | ------------- | ------------- | ------------- | ------------- | ------------- | ------------- | ------------- | ------------- | ------------- | ------------- | 58.17±7.14 | 38.42±2.39 | 2063±292 | 180.76±16.33 | 1.60±0.19 |
| 7. | Blocky polyhedral | 56.19±5.86 | 45.05±5.72 | 2496.52±554.77 | 187.63±20.23 | 1.26±0.062 | ------------- | ------------- | ------------- | ------------- | ------------- | ------------- | ------------- | ------------- | ------------- | ------------- | 49.97±1.20 | 33.39±2.81 | 1490±165 | 153.53±6.72 | 1.56±0.12 |
| 8. | Blocky rectangular | ------------- | ------------- | ------------- | ------------- | ------------- | ------------- | ------------- | ------------- | ------------- | ------------- | 128.47±15.94 | 33.17±5.15 | 4925±1050 | 334.5±47.49 | 3.83±0.23 | ------------- | ------------- | ------------- | ------------- | ------------- |
| 9. | Clavate | 26.39±0.91 | 11.51±1.69 | 317.73±51.55 | 79.62±4.39 | 1.77±0.26 | ------------- | ------------- | ------------- | ------------- | ------------- | ------------- | ------------- | ------------- | ------------- | ------------- | ------------- | ------------- | ------------- | ------------- | ------------- |
| 10. | Cross | ------------- | ------------- | ------------- | ------------- | ------------- | 17.55±1.18 | 15.34±1.22 | 217.25±24.20 | 77.24±5.27 | 1.30±0.04 | ------------- | ------------- | ------------- | ------------- | ------------- | ------------- | ------------- | ------------- | ------------- | ------------- |
| 11. | Cuneiform bulliform | ------------- | ------------- | ------------- | ------------- | ------------- | ------------- | ------------- | ------------- | ------------- | ------------- | 60.98±6.58 | 40.01±5.34 | 2032.51±555 | 182.4±21.89 | 1.36±0.01 | ------------- | ------------- | ------------- | ------------- | ------------- |
| 12. | Cylindrical | ------------- | ------------- | ------------- | ------------- | ------------- | ------------- | ------------- | ------------- | ------------- | ------------- | 120.60±5.86 | 8.63±1.38 | 1060±152.28 | 259.49±11.94 | 13.33±2.1 | ------------- | ------------- | ------------- | ------------- | ------------- |
| 13. | Elongate dentate | ------------- | ------------- | ------------- | ------------- | ------------- | 109.07±13.93 | 13.15±1.23 | 1863±477.34 | 278.92±33.89 | 6.77±0.23 | 87.24±13.75 | 10.76±0.65 | 1018±170 | 212,5±29.94 | 7.21±1.28 | ------------- | ------------- | ------------- | ------------- | ------------- |
| 14. | Elongate ridged | 109.56±12.99 | 8.82±0.66 | 897±71.77 | 241.8±26.27 | 12.10±1.87 | ------------- | ------------- | ------------- | ------------- | ------------- | ------------- | ------------- | ------------- | ------------- | ------------- | ------------- | ------------- | ------------- | ------------- | ------------- |
| 15. | Elongate smooth | ------------- | ------------- | ------------- | ------------- | ------------- | 143.73±24.17 | 10.09±0.82 | 1618.4±462.6 | 308.5±49.05 | 13.21±1.98 | 87.48±14.48 | 3.39±0.25 | 387.45±70.20 | 186.24±29.45 | 17.13±2.9 | ------------- | ------------- | ------------- | ------------- | ------------- |
| 16. | Elongate Trachied | 77.89±6.01 | 47.83±6.39 | 3260.6±291 | 237.86±10.96 | 1.88±0.39 | ------------- | ------------- | ------------- | ------------- | ------------- | ------------- | ------------- | ------------- | ------------- | ------------- | ------------- | ------------- | ------------- | ------------- | ------------- |
| 17. | Macrohair | ------------- | ------------- | ------------- | ------------- | ------------- | ------------- | ------------- | ------------- | ------------- | ------------- | ------------- | ------------- | ------------- | ------------- | ------------- | 124.99±16.5 | 21.77±2.32 | 2682±726 | 308±39.98 | 5.02±0.22 |
| 18. | M-shaped nodular bilobate | 27.94±2.49 | 13.43±1.39 | 306.67±31.56 | 90.89±5.04 | 2.55±0.57 | ------------- | ------------- | ------------- | ------------- | ------------- | 21.05±1.78 | 9.94±0.79 | 204.40±25.16 | 71.52±4.53 | 2.31±0.33 | ------------- | ------------- | ------------- | ------------- | ------------- |
| 19. | Nodular bilobate | ------------- | ------------- | ------------- | ------------- | ------------- | 23.75±2.23 | 8.29±0.67 | 209.77±34.73 | 68.75±4.52 | 2.79±0.41 | 26.65±1.56 | 8.37±0.41 | 190.16±19.50 | 74.76±3.53 | 3.61±0.25 | 20.22±1.55 | 8.06±0.89 | 153.04±13.09 | 61.17±2.72 | 2.39±0.33 |
| 20. | Orbicular | 40.19±7.03 | 35.90±5.79 | 1551.50±549.38 | 137.71±23.66 | 1.15±0.05 | ------------- | ------------- | ------------- | ------------- | ------------- | ------------- | ------------- | ------------- | ------------- | ------------- | ------------- | ------------- | ------------- | ------------- | ------------- |
| 21. | Parallepipedal | 50.49±8.88 | 25.93±6.02 | 1430.50±623 | 143.68±27.61 | 1.98±0.19 | 36.38±3.78 | 20.21±1.96 | 746.35±136 | 109±10.17 | 1.79±0.12 | 54.84±6.62 | 23.68±1.43 | 1381.5±319 | 165.58±23.86 | 2.28±0.20 | 36.24±4.44 | 16.48±4.49 | 700±243.4 | 106.2±16.17 | 2.46±0.49 |
| 22. | Parallepipedal bulliform cell | ------------- | ------------- | ------------- | ------------- | ------------- | ------------- | ------------- | ------------- | ------------- | ------------- | 51.05±5.74 | 21.36±2.21 | 1252±168 | 151.99±8.67 | 2.36±0.43 | ------------- | ------------- | ------------- | ------------- | ------------- |
| 23. | Polylobate | ------------- | ------------- | ------------- | ------------- | ------------- | ------------- | ------------- | ------------- | ------------- | ------------- | 34.38±1.98 | 9.35±0.60 | 284.11±33.82 | 94.09±5.90 | 4.03±0.26 | ------------- | ------------- | ------------- | ------------- | ------------- |
| 24. | Polylobate irregular | ------------- | ------------- | ------------- | ------------- | ------------- | ------------- | ------------- | ------------- | ------------- | ------------- | 32.03±1.48 | 8.21±0.79 | 233.18±32.12 | 83.14±3.52 | 4.31±0.38 | ------------- | ------------- | ------------- | ------------- | ------------- |
| 25. | Prickle hair | ------------- | ------------- | ------------- | ------------- | ------------- | ------------- | ------------- | ------------- | ------------- | ------------- | ------------- | ------------- | ------------- | ------------- | ------------- | 41.38±4.70 | 5.23±0.78 | 217.51±56.04 | 94.79±10.79 | 5.99±0.49 |
| 26. | Rondel (elongate/notched type) | 19.56±1.99 | 8.82±0.66 | 177.17±11.77 | 81.88±6.27 | 2.65±0.15 | ------------- | ------------- | ------------- | ------------- | ------------- | ------------- | ------------- | ------------- | ------------- | ------------- | ------------- | ------------- | ------------- | ------------- | ------------- |
| 27. | Rondelcomplex | ------------- | ------------- | ------------- | ------------- | ------------- | ------------- | ------------- | ------------- | ------------- | ------------- | ------------- | ------------- | ------------- | ------------- | ------------- | 44.03±5.15 | 18.99±3.90 | 889±214 | 127.59±15.72 | 2.50±0.41 |
| 28. | Saddle | ------------- | ------------- | ------------- | ------------- | ------------- | ------------- | ------------- | ------------- | ------------- | ------------- | ------------- | ------------- | ------------- | ------------- | ------------- | 11.87±0.38 | 7.67±0.36 | 92.77±8.67 | 40.55±0.86 | 1.52±0.06 |
| 29. | Saddle (narrow/deformed  type) | ------------- | ------------- | ------------- | ------------- | ------------- | 39.96±1.81 | 17.05±1.16 | 741.09±46.95 | 121.48±4.32 | 2.28±0.19 | ------------- | ------------- | ------------- | ------------- | ------------- | ------------- | ------------- | ------------- | ------------- | ------------- |
| 30. | Scutiform | ------------- | ------------- | ------------- | ------------- | ------------- | 37.96±7.22 | 13.80±2.77 | 499.38±235.5 | 98.58±21.25 | 2.42±0.27 | 31.37±4.70 | 20.94±2.69 | 635.21±194.5 | 101.86±14.3 | 1.43±0.07 | 37.61±3.54 | 21.50±1.24 | 877.14±145 | 129.05±15.16 | 1.77±0.19 |
| 31. | Spheroid irregular | ------------- | ------------- | ------------- | ------------- | ------------- | ------------- | ------------- | ------------- | ------------- | ------------- | ------------- | ------------- | ------------- | ------------- | ------------- | 36.49±4.29 | 32.15±2.62 | 1173±180 | 127±10.49 | 1.26±0.03 |
| 32. | *Stipa-* type bilobates | 24.47±2.50 | 8.34±0.49 | 175.79±7.71 | 64.36±3.30 | 3.23±0.39 | ------------- | ------------- | ------------- | ------------- | ------------- | 16.67±1.45 | 9.22±0.65 | 149.35±11.86 | 54.60±2.31 | 1.94±0.23 | ------------- | ------------- | ------------- | ------------- | ------------- |
| 33. | Stomata | ------------- | ------------- | ------------- | ------------- | ------------- | 29.77±1.77 | 19.73±1.19 | 525.46±40.69 | 86.14±3.37 | 1.27±0.06 | ------------- | ------------- | ------------- | ------------- | ------------- | ------------- | ------------- | ------------- | ------------- | ------------- |
| 34. | Tabular polyhedral | ------------- | ------------- | ------------- | ------------- | ------------- | 38.53±4.52 | 28.64±1.50 | 1202±239 | 141.83±19.37 | 1.59±0.19 | ------------- | ------------- | ------------- | ------------- | ------------- | 27.89±5.83 | 22.36±4.08 | 671.66±256 | 92.80±17.09 | 1.25±0.06 |
| 35. | Tabular tracheid | ------------- | ------------- | ------------- | ------------- | ------------- | 74.78±24.49 | 23.90±6.03 | 2522±675 | 204±55 | 2.91±0.54 | ------------- | ------------- | ------------- | ------------- | ------------- | ------------- | ------------- | ------------- | ------------- | ------------- |
| 36. | Tower flat | 19.11±2.58 | 15.69±1.97 | 341.27±60.89 | 72.48±6.40 | 1.21±0.017 | ------------- | ------------- | ------------- | ------------- | ------------- | ------------- | ------------- | ------------- | ------------- | ------------- | 17.23±2.68 | 12.34±0.89 | 201.25±44.93 | 59.49±5.88 | 1.53±0.08 |
| 37. | Trapezoid | ------------- | ------------- | ------------- | ------------- | ------------- | 55.24±3.54 | 29.34±2.22 | 1597.71±145 | 172±8.99 | 1.80±0.15 | 32.21±2.7 | 26.20±2.34 | 985.52±148 | 123.43±9.11 | 1.21±0.05 | ------------- | ------------- | ------------- | ------------- | ------------- |
| 38. | Trapezoidal keeled tracheid | ------------- | ------------- | ------------- | ------------- | ------------- | 93.64±11.93 | 18.60±3.01 | 2333±546 | 256±33.75 | 3.05±0.44 | ------------- | ------------- | ------------- | ------------- | ------------- | ------------- | ------------- | ------------- | ------------- | ------------- |

The entries in the Table indicate Mean±SE &[-------]= Absence of phytolith morphotype; A/R=Aspect ratio & Prem= Perimeter
